# A Complex Small RNA Repertoire Is Generated by a Plant/Fungal-Like Machinery and Effected by a Metazoan-Like Argonaute in the Single-Cell Human Parasite *Toxoplasma gondii*

**DOI:** 10.1371/journal.ppat.1000920

**Published:** 2010-05-27

**Authors:** Laurence Braun, Dominique Cannella, Philippe Ortet, Mohamed Barakat, Céline F. Sautel, Sylvie Kieffer, Jérôme Garin, Olivier Bastien, Olivier Voinnet, Mohamed-Ali Hakimi

**Affiliations:** 1 Laboratoire Adaptation et Pathogénie des Micro-organismes, CNRS UMR 5163 – ATIP+ group, Université Joseph Fourier, Grenoble, France; 2 CEA, DSV, IBEB, LEMiRE, CNRS, Université Aix-Marseille II, CEA Cadarache, Saint-Paul-lez-Durance, France; 3 DSV/IRTSV Laboratoire EdyP, CEA, Grenoble, France; 4 UMR 5168 CNRS-CEA-INRA- Joseph Fourier University; CEA Grenoble, France; 5 Institut de Biologie Moléculaire des Plantes du CNRS, Université de Strasbourg, Strasbourg, France; University of California Riverside, United States of America

## Abstract

In RNA silencing, small RNAs produced by the RNase-III Dicer guide Argonaute-like proteins as part of RNA-induced silencing complexes (RISC) to regulate gene expression transcriptionally or post-transcriptionally. Here, we have characterized the RNA silencing machinery and exhaustive small RNAome of *Toxoplasma gondii*, member of the Apicomplexa, a phylum of animal- and human-infecting parasites that cause extensive health and economic damages to human populations worldwide. Remarkably, the small RNA-generating machinery of *Toxoplasma* is phylogenetically and functionally related to that of plants and fungi, and accounts for an exceptionally diverse array of small RNAs. This array includes conspicuous populations of repeat-associated small interfering RNA (siRNA), which, as in plants, likely generate and maintain heterochromatin at DNA repeats and satellites. *Toxoplasma* small RNAs also include many microRNAs with clear metazoan-like features whose accumulation is sometimes extremely high and dynamic, an unexpected finding given that *Toxoplasma* is a unicellular protist. Both plant-like heterochromatic small RNAs and metazoan-like microRNAs bind to a single Argonaute protein, *Tg*-AGO. *Toxoplasma* miRNAs co-sediment with polyribosomes, and thus, are likely to act as translational regulators, consistent with the lack of catalytic residues in *Tg*-AGO. Mass spectrometric analyses of the *Tg*-AGO protein complex revealed a common set of virtually all known RISC components so far characterized in human and Drosophila, as well as novel proteins involved in RNA metabolism. In agreement with its loading with heterochromatic small RNAs, *Tg*-AGO also associates substoichiometrically with components of known chromatin-repressing complexes. Thus, a puzzling patchwork of silencing processor and effector proteins from plant, fungal and metazoan origin accounts for the production and action of an unsuspected variety of small RNAs in the single-cell parasite *Toxoplasma* and possibly in other apicomplexans. This study establishes *Toxoplasma* as a unique model system for studying the evolution and molecular mechanisms of RNA silencing among eukaryotes.

## Author Summary

*Toxoplasma gondii* is an important human parasite that causes life-threatening diseases in developing fetuses and in immunocompromised individuals, especially AIDS and transplant patients. Curiously, the *Toxoplasma* genome is deprived of most of the basic transcription factors that regulate gene expression in other eukaryotic cells. Therefore, alternative strategies must exist to modulate the many phases of the *Toxoplasma* complex life cycle that includes invasion of several hosts. Here, we investigate one of these strategies, by studying the repertoire of *Toxoplasma* silencing small RNAs (sRNAs). In eukaryotes, most of these regulatory molecules, 20–30nt-long, are produced by members of the Dicer RNase-III family, and exert their various functions through ubiquitous proteins called Argonaute (Ago). The surprising diversity of the *Toxoplasma* sRNAome uncovered in our study is consistent with those molecules exerting key functions during the parasite's life cycle, including, possibly, during virulent infection. The study also unravels an unsuspected level of complexity in the origin and mechanisms of action of the factors that generate and affect *Toxoplasma* sRNA, prompting a re-evaluation of our current views on RNA silencing in eukaryotes.

## Introduction

Apicomplexa are unicellular eukaryotes that multiply intracellularly in their mammalian hosts. They include parasites of major medical importance like *Plasmodium* species, the causative agent of malaria, and *Toxoplasma gondii*, the most widespread apicomplexan parasite, present virtually everywhere on earth. Although usually causing only mild symptoms in the adult, *Toxoplasma* can cause severe and life-threatening diseases in developing fetuses and in immunocompromised individuals, especially AIDS and transplant patients [1], [2]. *Toxoplasma* has a complex life cycle that includes infections of more than one host organism, differentiation through several morphologically distinct forms, and both sexual and asexual replication [3]. Changes in gene expression is expected as (i) parasites progress through the cell cycle, (ii) parasites differentiate in specific stages, and (iii) parasites are exposed to the host immune system during infection [4]. How these changes are regulated at the molecular level remains to a large extent unknown. A puzzling feature is the apparent lack, in apicomplexan parasites, of large families of recognizable specific transcription factors (TFs) operating in other eukaryotes [5]. Despite the paucity of recognizable TFs, apicomplexans are endowed with a rich repertoire of enzymes associated with epigenetics and chromatin remodeling, and this observation has fueled the idea that epigenetics could play an important role in the control of gene expression [6], [7].

Small regulatory RNAs are linked to epigenetic regulation of gene expression in several organisms but these are presently understudied in the Apicomplexa. The defining features of small silencing RNAs are their short length (∼20–30 nucleotides) and their association with members of the Piwi/Argonaute (AGO) family of proteins, which they guide to their regulatory targets [8], [9]. Many, albeit not all, small RNAs (sRNA) are produced by the RNase III-related enzyme Dicer. Small interfering RNAs (siRNA) are generated as populations from multiple Dicer cleavages along long dsRNA precursors, whereas microRNAs (miRNA) are discrete species generated from a single Dicer cleavage event of noncoding primary precursor transcripts containing small, imperfect stem–loop structures [10]. These distinct small RNA pathways compete and collaborate as they regulate genes and protect genome integrity from invading nucleic acids including viruses and transposons. They function as guides for effector complexes (RNA-induced silencing complexes, RISCs) that regulate gene expression by degrading mRNA, repressing its translation, or modifying chromatin. RNA silencing is an evolutionary ancient regulatory mechanism, and small RNA pathways in unicellular organisms appear, so far, to be relatively simple. In fission yeast, a single class of endogenous siRNAs has demonstrated roles in epigenetic silencing at centromeres and the initiation of heterochromatin assembly at the *mat* locus [11]. In the ciliated protozoan *Tetrahymena thermophila*, small RNAs are involved in developmentally regulated DNA elimination [12], [13] and post-transcriptional gene regulation [14]. Particularly surprising is the recent finding that the unicellular green alga *Chlamydomonas* produces microRNAs that had been previously associated with developmental regulation and multi-cellularity [15], [16].

Here, we show that the *T. gondii* genome, unlike in *Plasmodium* species [17], encodes all core components of an elaborate RNA silencing machinery that has been evolutionary shaped as a patchwork of factors of plant and fungal origin. We establish a comprehensive sRNA landscape of *T. gondii* through deep sequencing, and unravel that the most abundant sRNA classes are formed by metazoan-like miRNAs as well as plant-like repeat-and-satellite-associated sRNAs coined rdsRNA and satRNA, respectively. Beyond the surprising complexity of the small RNAome, we provide a thorough biochemical characterization of the proteins that associate with the single *T. gondii* Argonaute protein, *Tg*-AGO. Unexpectedly these proteins constitute the near-entire cohort of previously identified human and fly miRNA-RISC components. Our data indicate that miRNA-loaded *T. gondii* argonaute associates with polysome probably to regulate translation of *Tg*-miRNA predicted targets, many of which include mRNA with perfect or near perfect complementarity. *Tg*-AGO also co-purifies with chromatin-repressing complexes, suggesting a role in transcriptional silencing, most likely through it demonstrated association with rdsRNAs or satRNAs.

## Results/Discussion

### The *Toxoplasma* RNA silencing machinery

**Figure 1:**
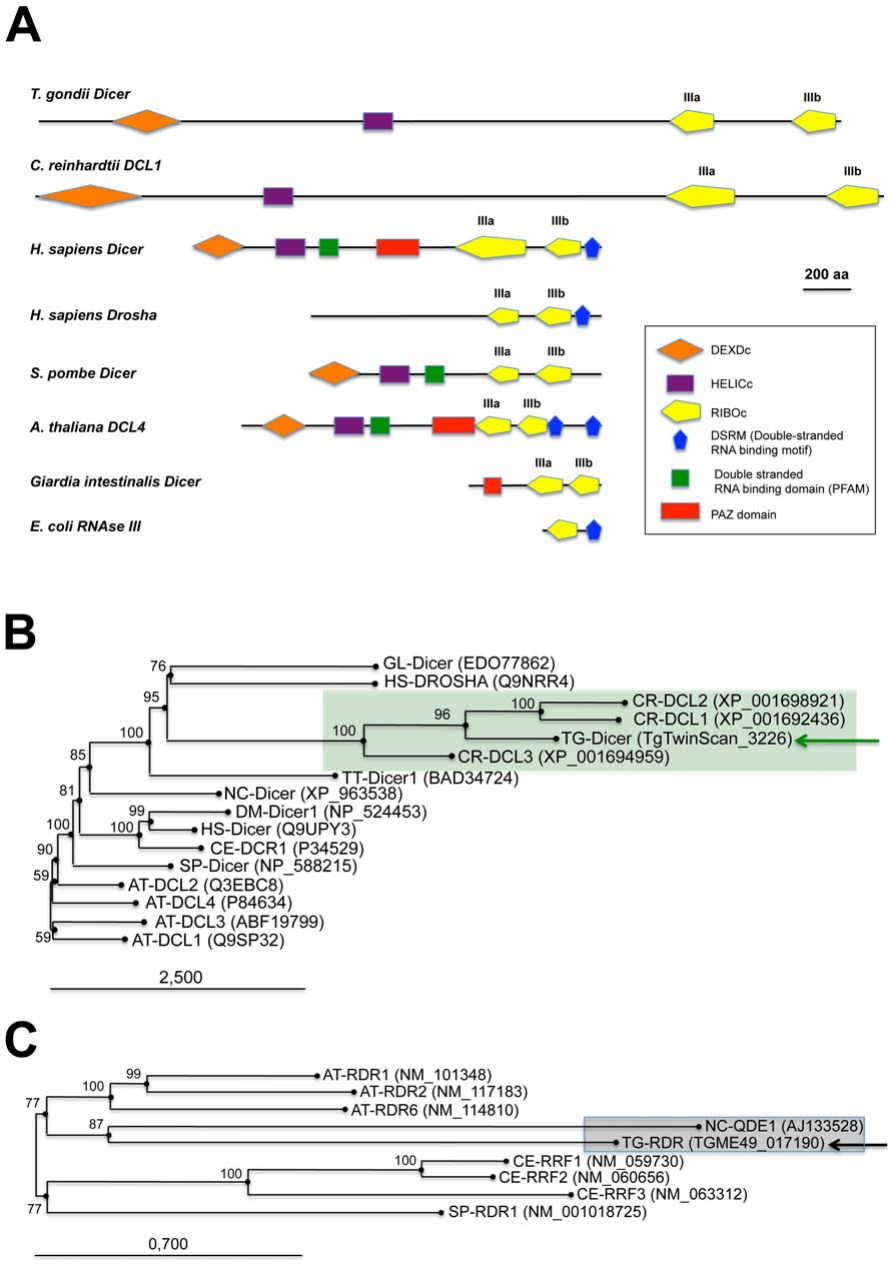
Domain organization and phylogenetic analysis of Dicer and RDR proteins. (A) Dicer proteins cleave dsRNA precursors into characteristic lengths through the action of two RNase III domains (in yellow). The domain arrangement of several Dicer enzymes is compared to *Toxoplasma Tg*-Dicer (TgTwinScan_3226, TOXODB v4.3). Evolutionary relationships between Dicer (B) and RDR (C) proteins from the following species: AT: *Arabidopsis thaliana*, GL: *Giardia lambdia*, SP: *Schizosaccharomyces pombe*, DM: *Drosophila melanogaster*, HS: *Homo sapiens*, CE: *Caenorhabditis elegans*, CR: *Chlamydomonas reinhardtii*, NC: *Neurospora crassa*, TT: *Tetrahymena thermophila*, TG: *Toxoplasma gondii*. *Tg*-Dicer and *Tg*-RDR are shown by a green and black arrows respectively and their branch family are shaded in green and gray. Numbers above the nodes indicate neighbor-joining bootstrap percentages.

Previous analyses have suggested a monophyletic origin for plant and animal Dicer proteins [18]. Sequence analyses show that the *Toxoplasma* genome (TOXODB release v6.0) [19] encodes only one Dicer-like protein, *Tg-*Dicer, which displays significant variability in primary sequence and domain organization compared to the Dicer consensus (Figure 1A): *Tg-*Dicer possesses an RNA helicase domain and two RNaseIII catalytic domains (RNaseIIIa and RNaseIIIb), but it lacks recognizable domains for dsRNA binding (DSRM) and PIWI-ARGONAUTE-ZWILLE (PAZ) functions. This organization is strikingly reminiscent of the DCL1 protein of the single cell algae *C. reinhardtii* (Figure 1A). *Toxoplasma* and *Chlamydomonas* Dicer-like sequences seem, indeed, orthologous, as they form a specific clade supported by a strong bootstrap score (Figure 1B), a consequence of a Drosha-like signature polypeptide that is more related (albeit weakly) to eubacterial RNaseIII enzymes and known to form an out-group with respect to higher plant and animal Dicers [18].

**Figure 2:**
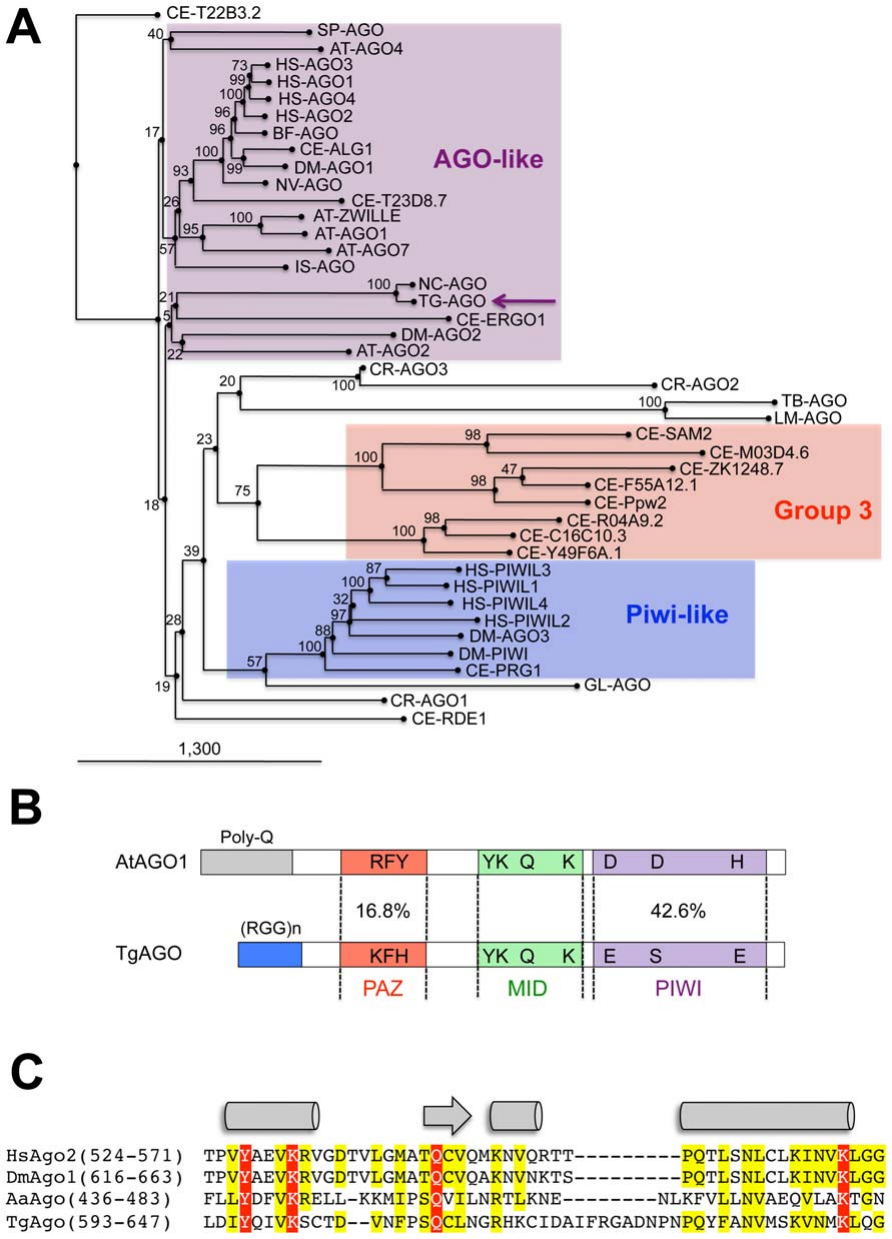
Domain organization and phylogenetic analysis of Argonaute and PIWI proteins. (A) Evolutionary relationships between Argonaute and Piwi proteins from the following species: AT: *Arabidopsis thaliana*, GL: *Giardia lambdia*, SP: *Schizosaccharomyces pombe*, DM: *Drosophila melanogaster*, HS: *Homo sapiens*, CE: *Caenorhabditis elegans*, CR: *Chlamydomonas reinhardtii*, NC: *Neospora caninum*, TT: *Tetrahymena thermophila*, TG: *Toxoplasma gondii*. *Tg*-AGO (accession number: GU046561) is pointed out by a pink arrow. Argonaute (in pink), Piwi (in blue) and group 3 (in red) branch family are represented as shaded boxes. Numbers above the nodes indicate neighbor-joining bootstrap percentages. (B) Schematic depiction of *Toxoplasma Tg-*AGO and *Arabidopsis At*-AGO1 (GeneID: 841262) domain according to archaeon Aquifex aeolicus X-ray crystal structure. The amino-terminal domain (grey or blue) is linked to the PAZ domain (red). The MID domain (green) connects the PAZ domain with the PIWI domain (pink) at the carboxy-terminal end of the protein. In the PAZ domain, residues important for binding of small RNA 3′ ends are indicated. In the MID domain, the residues required for 5′ end binding to small RNAs and binding to the 7-methylguanine (m7G) cap of target mRNAs are shown. The conserved residues for RNase H cleavage activity (DDH) in the PIWI domain are also shown. RGG indicates the domain of *Tg-*AGO containing arginine-glycine-glycine repeats. Poly Q, polyglutamine-containing domain in *Arabidopsis* Ago1. (C) Sequence alignment of conserved 5′–phosphate binding residues (MID domain) across various species of Argonaute proteins. Prefix Hs, *Homo sapiens*; Dm, *Drosophila melanogaster*; Aa, *Aquifex aeolicus*. Conserved residues are shaded in red.

Ago-related proteins are divided into the Ago-like and Piwi-like subfamilies (Figure 2A); a third clade, termed ‘Group 3 Argonautes’, is worm-specific [20]. *Toxoplasma* Argonaute (*Tg-*AGO) is represented at a single genomic locus; there is no evidence for *Toxoplasma*-encoded Piwi proteins. *Tg-*AGO belongs to the Ago-like family but only with weak bootstrap support, suggesting that the protein diverges significantly from its metazoan and plant counterparts. Nonetheless, the two key signature domains of the AGO family —the PAZ domain and the C-terminal PIWI domain— are conserved in *Tg-*AGO (Figure 2B). Overall, the PIWI domain shows the highest degree of conservation, but the Asp-Asp-Glu/Asp catalytic triad required for slicer activity is not found, suggesting that the protein lacks endonucleolytic cleavage capacity (Figure 2B). The second signature motif, the PAZ domain, contains only a few residues that are strictly conserved, while the middle (MID) domain of *Tg-*AGO harbors the residues (Y596, K600, Q610 and K644; Figure 2C) required to bind the characteristic 5′ phosphate group of guide small RNA strands [21], [22]. We also noted the presence, in the amino terminal part of *Tg-*AGO, of a stretch of repeated RGG residues (amino acids 1–68), in which the arginines have the potential to undergo methylation (Figure 2B). This feature is found in metazoan and plant AGO-related proteins and was shown to alter their stability and/or sub-cellular distribution, ultimately impacting their function [23], [24], [25].

A phylogenetic tree constructed by aligning the stereotypical RNA-dependent RNA polymerase (RDR) domain supports the monophyletic origin of the proteins found in *C. elegans*, fungi and *Arabidopsis* [26]. Inspection of the *Toxoplasma* genome showed the presence of a single RDR-like gene, suggesting the existence of an amplified RNA silencing machinery in this organism. *Tg-*RDR is closely related to *Neurospora crassa* RDR, QDE1, and forms a specific clade with plant RDRs, which itself constitutes an out-group from the RDRs of metazoans and from the fission yeast *S. pombe* (Figure 1C). We conclude from this analysis that a patchwork of factors of plant and fungal origin form the core processor components of the *Toxoplasma* RNA silencing machinery. This finding can be rationalized partly by the fact that the apicomplexa ancestor is a presumed endosymbiont of red algae [27]. We note, however, the moderate or poor phylogenetic relationship observed between *Tg-*Dicer, *Tg-*AGO and the corresponding paralogous proteins of its mammalian hosts.

### Complexity of the *Toxoplasma* small RNAome

**Figure 3:**
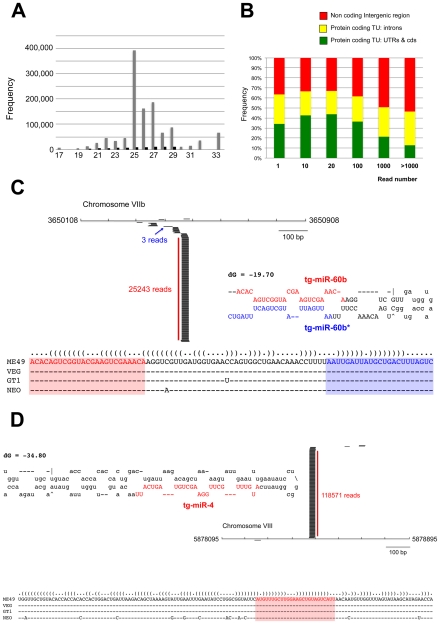
A repertoire of *Toxoplasma* endogenous small RNAs. (A) Size distribution of *Toxoplasma* sRNAs. The sets of redundant (grey) and unique (black) small RNAs were used to generate a histogram quantifying the number of sequences obtained for each size class. nt., nucleotides. (B) Distribution of small RNAs across the *Toxoplasma* genome. TU: Transcriptional unit, CDS: CoDing Sequence, UTRs: UnTranslated Regions. (C) A miR-60b production hot spot in chromosome VIIb is shown. Small RNAs with perfect matches were plotted within a 800-bp sliding window. Short thin lines above the long bars represent small RNAs derived from the antisense strands, and lines below the bars represent small RNAs from the sense strands. Vertical bars represent the consensus positions of sequencing reads that mapped to the predicted precursors, and the values indicate the total number of these reads. The fold-back structure of the precursor predicted with mfold is provided below. The mature region is shown in red and the passenger strand (microRNA*) in blue. The miR-60b–containing locus for the three canonical strains of *Toxoplasma* and *Neospora caninum* is depicted by sequence and structure. (D) A miR-4 production hot spot in chromosome VIII is shown along with the predicted structure and the sequence conservation across parasite species.

Having established that the *Toxoplasma* genome encodes all core components of an elaborate RNA silencing machinery, we sought to determine the small RNA landscape of this organism. To this aim, we prepared total RNA from freshly released, filtered parasites. Ethidium bromide staining revealed a relatively abundant class of small (s)RNAs, ∼30 nucleotides (nt) in length (Figure S1A). The 20–40nt sRNA fraction was recovered by gel excision, cloned and subjected to deep sequencing using the *Illumina* technology. The sRNA library was constructed so as to represent only those sRNAs with a 5′ monophosphate and a 3′ hydroxyl group, the termini expected of miRNAs and siRNAs [28]. About 75% of a total of 5,701,506 reads, represented tRNA and rRNA turnover products, as previously reported for other organisms (Figure S1B) [29]. After filtering low quality reads, 3′, 5′ adapters, and reads shorter than 17 nucleotides, a remaining 1,555,290 reads (∼30% of total reads) matched the *Toxoplasma* genome (ToxoDB, version 4.3) (Figure S1B). Comparing mRNA and sRNA data for highly expressed genes suggested that the sRNA fraction contained only a very low level of degradation products from longer mRNAs (data not shown). Most sRNA sequences were found to be 25–27nt in length, with 25nt representing the dominant size class (Figure 3A). Of the 1,222,203 total reads, 94,170 corresponded to non-redundant sRNAs, and 92% of these were single reads, thus unraveling a highly complex sRNA population. Plotting *Toxoplasma* sRNAs (*Tg*-sRNAs) species with 100% match on the reference genome (in 10-kb sliding windows) showed that non-redundant *Tg*-sRNA with high read numbers (>1000) originate predominantly from non-coding intergenic regions or are embedded within introns of protein-coding transcriptional units (TUs) (Figure 3B). Other, medium-to-low abundance *Tg*-sRNA, by contrast, mapped to protein-coding TUs and a variety of DNA repeats and satellites (Figure 3B). These two classes of *Tg*-sRNA were detailed further, as described in the following sections.

### *Toxoplasma* miRNAs

**Figure 4:**
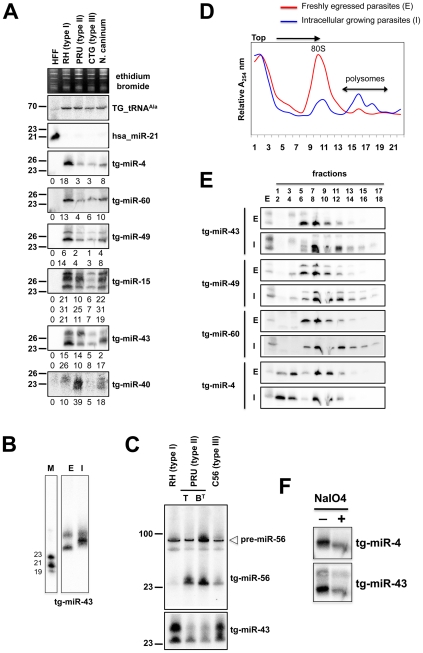
Expression patterns and characteristics of representative microRNAs in *Toxoplasma.* (A) Expression profile analyses of six parasite microRNA candidates by small-RNA Northern hybridization in three canonical strains of *Toxoplasma* and its close relative, *Neospora caninum*. To control for loading, blots were stripped and rehybridized with the indicated miRNA and the control probes (tRNA^Ala^ and hsa_miR-21). Ethidium bromide staining of rRNA was used as loading control. Hybridization signals were quantified with the ImageJ software, normalized to the amount of *Toxoplasma* tRNA^Ala^ signal present in each sample, and shown as digit below each Northern blot. For miR-15, -43 and -49, each individual signal was separately quantified. The number next to each panel represents the position of RNA markers. Tg, *Toxoplasma gondii*; hsa, *Homo sapiens*. (B) Total RNA from freshly egressed (E) and intracellular growing parasites (I) were analyzed by Northern hybridization with complementary probes for *Tg*-miR-43. RNA markers (left lane) are 19, 21 and 23 nucleotides. (C) Total RNA from types I, II (tachyzoite and pH-converted bradyzoite) and III were analyzed by Northern hybridization with complementary probes for *Tg*-miR-43 and -miR-56. Pre-miR indicates the foldback RNA precursor form; *Tg*-miR refers to the mature, predominant form. The number next to each panel represents the position of RNA markers. (D) Total RNA from freshly egressed (E, in red) and intracellular growing parasites (I, in blue) were subjected to sedimentation on 5%–45% sucrose gradients in the presence of cycloheximide (to preserve the association of translating ribosomes with mRNAs). Measurements of the total RNA in the gradient fractions reveal signature patterns of peaks corresponding to messenger-ribonucleoprotein complexes (mRNP), followed by the 80S ribosomal fractions and the polyribosomal fraction. As can be appreciated from the A254 profiles, the rapid proliferation of tachyzoites inside of the host cell (in blue) is diagnosed by the high level of translating polyribosomes. (E) A proportion of *Tg*-miRNAs cosediment with polyribosomes. Total RNA was extracted from each fraction and analyzed by Northern blots with complementary probes for 6 *Tg-*miRNAs [*miR*-*43*, -*49*, and *60* (shown) and *mir*-*4*, -*15*, and *40*, (not shown)]. All of the *Tg*-miRNAs tested are predominantly localized to the mRNP with a clear shift to polyribosome fractions in intracellular growing parasites. (F) The 3′ end of *Tg*-miR-4 and -43 are not modified. Gel-fractionated small RNAs were examined by b-elimination assay (see methods).

In a search for putative *Toxoplasma* miRNA candidates, we evaluated sRNA reads exhibiting the following features: (1) high abundance of sequence reads sharing the same 5′ terminus; (2) exact match to one or several genomic loci displaying a characteristic fold-back structure typical of *MIRNA* precursors; and (3), when applicable, low abundant sequence reads corresponding to the labile (miRNA*) passenger strand of miRNA/miRNA* duplexes, as predicted within the fold back structures. Fourteen sRNA families cloned at a high frequency met these *MIRNA* features and were thus annotated with high confidence as *T. gondii* miRNAs (*Tg-*miRNAs) (Figures 3, S2-S13 and Table S1). Genome browser views of some of these *Tg-MIRNA* indeed indicated the existence of a low frequency, single miRNA* (passenger strand) corresponding to the opposite strand of the duplex within the fold back-structure (e.g. Tg-miR-60b; Figure 3C). Moreover, the reconstituted duplexes sometimes had small 3′ overhangs characteristic of Dicer processing (Figures 3C, S8-S10). Of the 14 annotated *Tg*-miRNA families, 7 gave detectable hybridization signals in Northern analysis carried out with 5′ end-labeled antisense oligonucleotides (Figures 4A, 4C and S14). No signal was detected with RNA extracted from host cells, confirming that these sRNAs are *Toxoplasma*-specific. Hybridization often unraveled discrete sRNA species that were heterogeneous in size, reminiscent of the sRNA signals observed in *S. pombe* [30] and other organisms in which Dicer lacks a PAZ domain, required for the precise sizing of processed sRNAs [31], [32], [33]. In all cases, hybridization with antisense probes from precursor sequences flanking the mature miRNA gave no signal (data not shown), confirming the excision, by *Tg-*Dicer, of a single sRNA species, a landmark of plant and metazoan miRNA biogenesis. The members of the remaining 7 miRNA families were below detection levels, in agreement with their much lower cloning/sequencing frequencies (Figures S8-S13). In all cases -and as expected from their poor read counts due to their intrinsic instability- cloned miRNA* were also below detection levels of Northern analysis.

Sequencing and Northern analyses showed that the miR-60 family largely dominates the *Toxoplasma* miRNA landscape, accounting for 335,014 reads, of which 61% (280,723 reads) were contributed by miR-60a alone (Figure S2B). *MIR-60*, together with *MIR-4*, also constitute the two most diversified *Tg-MIRNA* gene families (with 8 distinct members in each) among the 14 families identified with high confidence (Figure S2B and S3B). In most cases of *Tg*-miRNAs with multiple precursors (6 families, Table S1), the mature miRNAs were not located on the same fold-back arms, which, furthermore, were also found to vary in sequence, suggesting that these genes do not share a common ancestor and, thus, have evolved separately. The 14 high-confidence *Tg*-miRNAs showed no significant homology to any of the known miRNAs of plants and metazoans, as assessed in the central miRBase depositary (release 14). Nearly all *Tg*-miRNAs and *Tg*-miRNA* (when available) had, however, directly identifiable orthologs in the genome of the apicomplexan *Neospora caninum* (dog parasite), when up to 3-nucleotide polymorphism was tolerated (Figures 3, S2-S8 and S13). Moreover, the size and abundance of these orthologous *N. caninum* (*Nc*)-miRNAs was confirmed by Northern blot analysis using the same antisense oligonucleotide probes employed for detection of *Tg-*miRNAs (Figures 4A and S14). The notable exception to sequence conservation in *N. caninum* was observed with *Tg*-miR-62, -64, -65 or -66, although this could be attributed to the incomplete *N. caninum* genome annotation (Figures S9-S12). Taking into account recent observations made in the single cell algae *Chlamydomonas*, apicomplexans thus provide the second reported example of unicellular organisms that produce miRNAs. Unlike in *Chlamydomonas*, nonetheless, and despite the relatedness of the Dicer proteins found in both organisms (Figure 1A and 1B), *Tg-MIRNA* fold-backs have length and thermodynamic features that are much closer to those of mammalian hosts than those of *Chlamydomonas* or higher plants [10]. Consistent with this idea, most *Tg*-miRNAs display a clear 5′- nucleotide bias towards A, as also observed for most mammalian miRNAs [34]. Unlike many mammalian *MIRNAs*, however, *Tg*-*MIRNAs* were not found to form genomic clusters. These findings further emphasize the surprising mosaic nature of the *Toxoplasma* RNA silencing machinery and small RNA loci.

The above 14 miRNA families were identified through deep-sequencing of small RNAs isolated from freshly egressed parasites, and so other miRNAs might exist that were simply too low in abundance to be cloned under these specific growth conditions. In addition, several *Tg*-sRNAs cloned at moderate to low frequency mapped to imperfect fold-backs scattered along the genome, with relatively low free energy (Figure S15). These hairpins are much more heterogeneous in size and structure than cognate *Tg-MIRNA* precursors, yet their processing produces discrete sRNA species. Although their relatively modest cloning frequencies precludes their detection by Northern analysis, including in *N. caninum,* the corresponding sRNA might represent recently-evolved miRNAs that may engage into miRNA-like regulatory activities. In plants, a model for *MIRNA* gene evolution, termed “spontaneous evolution” stems from the high density of small-to-medium sized fold-back sequences scattered throughout the *Arabidopsis* genome. It has been proposed that following the capture of transcriptional regulatory sequences, some of these random fold-backs could occasionally give rise to new *MIR* genes. Stabilization through co-evolution with targets initially found by chance could then lead to the fixation of these genes in the genome [35].

### Biochemical properties of *Toxoplasma* miRNA

Unlike metazoan miRNAs, plant miRNAs are methylated at the 2′ hydroxy positions of their 3′-last nucleotides [36]. This modification, mediated by the methyl-transferase HEN1, protects miRNA from 3′ end uridylation and subsequent degradation [37]. However, *Tg*-miRNA species were found sensitive to b-elimination by periodate, which causes a diagnostic shift in sRNA mobility (Figure 4F). Thus, unlike their plant counterparts, but similar to metazoan miRNAs, *Tg*-miRNAs do not carry 3′-end modifications, a result also consistent with our failure to identify a HEN1 homolog in the *Toxoplasma* genome (TOXODB, release v5.2). Nonetheless, the 3′ end of several cloned *Tg*-miRNAs was often found to contain untemplated adenine residues, which must be added, therefore, after processing by an as yet unidentified terminal adenyl-transferase (Figure S3B). It was shown recently that addition of adenylic acid residues on the 3′-end apparently slows down miRNA turnover in *Populus trichocarpa* [38].

In plants and animals, miRNA are recruited by AGO proteins to enhance the turnover, or inhibit the translation of cognate mRNA targets. Consequently fractions of most plant and metazoan miRNAs associate with polysomes, the sites of active translation. The existence of miRNAs in *Toxoplasma* together with the absence of detectable slicer residues in the *Tg*-AGO predicted that *Tg*-miRNA would also associate, at least partly, with polysomes. To test this idea, protein extracts from freshly egressed parasites (E) ready to invade, or from fast-growing intracellular parasites (I), were fractionated and resolved on sucrose density gradients (see *Methods*). For the former (I), the absorbance profiles at 254 nM reflected the ribosome pattern expected from rapidly growing cells: there were few monosomes (80S) and the bulk of the ribosomes sedimented in the polysomal fractions (Figure 4D). By contrast, the amount of polyribosomes in invading parasites (E) was substantially reduced, and this was accompanied by a concomitant increase in 80S monosomes (Figure 4D). As previously observed in plants and metazoans, *Tg-*miRNAs distribution was found to span a wide range of molecular weights across the gradient (Figure 4E) [39], [40], [41]. Nonetheless, a fraction of several *Tg*-miRNAs co-sedimented with polysomes (Figure 4E, fractions 13–18). Moreover, the association with translating ribosomes was more pronounced in exponentially growing parasites (I), as expected. Not all *Tg*-miRNA, however, were found associated to polysomes, and this was notably the case of *Tg-*miR-4 (Figure 4E). *Tg*-miR-4 and other non-polysomal miRNA may regulate target mRNAs at later stages of parasite differentiation (e. g. bradyzoite); alternatively, they might not be involved in translation control (data not shown), as has been recently shown for a class of *Arabidopsis* miRNA [41] that use cleavage-competent and/or cleavage-resistant target sites found in specific non-coding RNAs to initiate the production of trans-acting (tasi)RNAs via the action of RDR6 [42]. Whether tasiRNA exist in *Toxoplasma* is an interesting question for future experiments. In any case, our findings demonstrate that several *Tg*-miRNAs are present in the form of miRNPs in polyribosome-containing fractions where they are likely to negatively regulate translation of target transcripts.

### Complex regulation of *Toxoplasma* MIRNA gene expression

miRNAs orchestrate many biological functions and are notably involved in cell fate determination and/or integration of developmental or external stimuli. Plant and metazoan miRNA expression may thus vary greatly depending on growth conditions, changes in developmental stages or, in the case of parasites, changes in virulence. To investigate if, similarly, *Tg-*miRNA accumulation/processing is regulated differentially in *Toxoplasma*, we sampled miRNA from freshly egressed (E) or intracellular (I) parasites, as well as from three classical *Toxoplasma* isotypes. These isotypes are representative of the European and North American parasite population and correspond to three clonal lineages, designated type I, II and III, corresponding to reference strains RH, PRU and CTG, respectively [43]. These genotypes display contrasted virulence in mice: type I strains are lethal, whereas type II and III strains are hypo-virulent and typically establish chronic infections. There are additional phenotypic differences in migration, growth rate, and ability to convert from tachyzoite to the cyst-forming bradyzoite stage, notably [43].

For several of the *Tg*-miR detected by Northern analysis, we observed differential abundances between the *Toxoplasma* strains (Figure 4A and 4C). Thus, normalized to the *Tg*-tRNA^Ala^ signal, the miR-4 signal was six fold greater in type I than it was in types II and III (Figure 4A). Likewise *Tg*-miR-4, -49 and -60 were more abundant in type I strain, whereas *Tg*-miR-40 and -56 were clearly more abundant in type II. Further investigation of these variations in miR-56 levels showed that they were attributable to differences in miRNA processing rather than transcription, because similar levels of pre-miR-56 were observed among the three isotypes, in Northern analyses (Figure 4C). This result reinforces the growing view that *MIRNA* genes can undergo extensive post-transcriptional regulation through mechanisms that selectively affect pri-miRNA processing and/or pre-miRNA stabilization [44], as uncovered recently with interactions involving murine pre-Let-7, lin-28 [45], [46] and the RNA-binding protein KSRP [47], a homolog of which was indeed found associated with *Tg*-AGO (see following sections). These observations thus extend this concept to a single cell parasite; given the overall low genetic diversity among *Toxoplasma* isotypes [43], they further suggest that differential regulations of pathogen's miRNA repertoires might, indeed, influence virulence. Analyses of *Tg*-miR accumulation between freshly egressed (E) and intracellular (I) *Toxoplasma* revealed additional scope for modulation of mature miRNA levels between the two parasitic states. For instance, there was a clear mobility shift with miR-43, which is unlikely explained by changes in pre-miR-43 steady levels, but rather, by alternative Dicer-mediated processing events producing small RNA length variants or with modified termini (Figure 4B). Collectively, these observations unravel highly complex regulations of *Tg*-*MIRNA* gene expression, which might be used to refine the amplitude or regulatory outputs of target gene regulation during the parasite's multiple biological states.

### *Toxoplasma* miRNA exhibit perfect or near-perfect matches to many target transcripts

We then attempted to identify putative targets for representative members of the 14 unambiguous *Tg*-*MIRNA* families retrieved in this study. A bioinformatics approach was used to scan *Toxoplasma* transcripts for *Tg*-miRNA complementarity sites. Despite the resemblance of Tg-*MIRNA* and mammalian *MIRNA* genes, and the association of both types of molecules to polysomes, the absence of slicer residues in the *Tg*-AGO protein strongly suggests that the parasites' miRNA engage into a distinct type of pairing to their targets (Figure 2B). Hence, in the mammalian host, most AGO2-bound miRNAs exhibit only moderate pairing to their targets, notably through a stretch of 6–7 contiguous 5′ nucleotides known as ‘seed’, which is usually followed by several central mismatches that sterically hinder the RNAseH activity of AGO2 [48], [49]. This loose miRNA:target pairing, which is thought to favor translational repression over slicing, makes it difficult to predict mammalian miRNA targets using computer algorithms. These algorithms, moreover, are often biased towards 3′ UTRs, because these regions evolve much more rapidly than coding regions, and are, therefore, more prone to the identification of contiguous, 6–7nt seed-complementary sequences [50].

We found that most of the 14 *Tg*-miRNA analyzed have readily identifiable target sites in a variety of cellular transcripts (Table S2 and data not shown). Interestingly, these sites exhibit complete to near-complete complementarity to miRNAs -a feature of plant but not of metazoan miRNAs- and they are found in 5′-UTR, coding region and 3′-UTR, although there is a clear bias towards the latter region for most miRNA analyzed (Figure S16A and Table S2). Allowing up to 3 mismatches, more than 80 putative target transcripts were identified for miR-60a alone, the most abundantly sequenced *Tg*-miRNA. Using the same stringent parameters, an average of 25 cellular targets could be retrieved for each of the 14 *Tg*-miRNA (Figure S16A and Table S2). GO-term analysis of the putative *Tg*-miRNA target transcripts showed that they encompass virtually all known biological functions, with a somewhat stronger emphasis on translational control and cell cycle regulation, which might be expected for a single-celled, highly dividing parasite (Figure S16B). These predicted *Toxoplasma* miRNA:target interactions thus constitute an unprecedented situation in all eukaryotes studied so far, whereby a miRNA-loaded, slicer-deficient Ago (see later in the text) might regulate target gene expression, presumably at the translational level, through perfect or near-perfect binding sites that are predominantly –albeit not exclusively- located in 3′-UTRs. To test the possibility of target cleavage and degradation mediated by *Tg*-AGO, we examined the levels of mRNA predicted as strong targets of isotype-specific *Tg*-miRNAs (Table S2). Real time PCR analyses revealed little, if any changes in mRNA levels between type I and II isotypes, contrasting with the differential abundances of the corresponding Tg-miRNAs. This result corroborates partially the suggestion that *Tg*-AGO acts mainly as a translational regulator, which is also in agreement with the cellular factors found in association with Tg-AGO (see later in the text). Owing to the lack of available antibodies for predicted targets and our current inability to generate *Tg-Dicer* or *Tg-AGO* mutants, experimental validation of the above hypothesis will be part of future experiments.

To date, the use of RNAi for specific gene silencing has remained largely inconclusive in Toxoplasma. Many laboratories have attempted to use this tool to down-regulate gene expression but very few reports showed successful double-stranded RNA induced gene silencing and there is currently no evidence for the production of specific siRNA [51]. We note that the use of RNAi is normally expected to result in mRNA turnover, as in metazoans or plants. The nature of the *Tg*-AGO (slicer deficient) and its possible mode of operation through translational repression (with a usually modest output on gene expression in metazoans) is obviously one parameter that could explain the lack of significant levels of mRNA degradation upon RNAi treatments in this organism.

### *Toxoplasma* repeat-and satellite-associated sRNA

**Figure 5:**
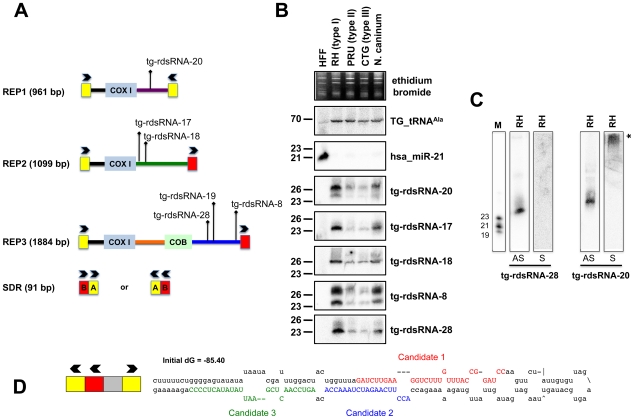
Expression patterns and characteristics of repeat-associated *Tg*-rdsRNA in *Toxoplasma*. (A) Schematic representation of REP elements. The three REP fragments and the short disperses repeat (SDR) are displayed from 5′ to 3′ with respect to the COX1 (cytochrome oxidase subunit 1) gene fragment. Blocks A and B of the SDR are labeled in yellow and red, respectively. Cloned *Tg*-rdsRNA are represented above: *Tg*-rdsRNA-8 (1155 reads), tg-rdsRNA-17 (464 reads), *Tg*-rdsRNA-18 (1134 reads), *Tg*-rdsRNA-19 (1326 reads), *Tg*-rdsRNA-20 (1066 reads) and *Tg*-rdsRNA-28 (223 reads). (B) Expression profile analyses of five parasite *Tg*-rdsRNA candidates by small RNA Northern hybridization in three canonical strains of *Toxoplasma* and its close relative, *N. caninum*. Controls and quantification method are as in Fig. 2A. The number next to each panel represents the position of RNA markers. (C) Small RNA blots were stripped and re-hybridized with antisense probes of selected *Tg*-rdsRNA sequence. Antisense and sense oligonucleotides (indicated by AS and S, respectively) were used to confirm the polarity of *Tg*-rdsRNA-20 and -28. (D) SDR-containing chromosomic region (TGME49_chrX:233424-233609) forms a stereotypic hairpin precursor predicted by mfold that harbor cloned small RNA candidates.

The largest bulk of medium-to-low abundance *Tg*-sRNAs does not meet the criteria of miRNA annotation and appears to match repetitive elements REP1, REP2 and REP3 (Figure 5A) [52]. REP elements are mitochondrial-like sequences dispersed throughout the nuclear genome of *Toxoplasma*. They are typically composed of mitochondrial-like genes, including COX1 (cytochrome oxidase subunit 1) and COB (apocytochrome b) that are flanked by a 91 bp short-dispersed repetitive sequence (SDR) organized as a direct or inverted repeat (Figure 5A) that might play roles in generation or dispersal of the REP elements. Nonetheless, there is no sequence similarity between SDRs and other terminal repeats such as those of retroviral LTRs. Moreover, REP elements do not seem to be highly mobile [52].

Genome mapping showed that *Toxoplasma* REP-derived sRNAs (rdsRNAs) form discrete species that are exclusively generated from regions located downstream of the COX1 and COB sequences, (Figure 5A), with read counts typically ranging from >10,000 (rdsRNA-17) to a few hundred reads (rdsRNA-28). This fairly high abundance might be explained by the fact that the estimated number of REP elements is >500 copies per genome [52]. While their size range (21–27nt, Table S3) and sensitivity to periodate (not shown) was similar to that of *Tg*-miRNAs, about half of the rdsRNAs had a 5′ terminal U instead of the prevalent A found in miRNAs. All tested *T*g-rdsRNAs were readily detected by Northern blotting using 5′end-labeled antisense oligonucleotides but, unlike *Tg*-miRNAs, they were consistently much more abundant in the highly virulent *Toxoplasma* isotype-I (Figure 5B). Interestingly, sense probes generated no signal for the *Tg*-rdsRNAs tested, consistent with the absence of *Illumina* reads corresponding to opposite-strand sRNA species (Figure 5C and data not shown). A sense probe for *Tg-*rdsRNA-20, however, gave a high molecular weight signal potentially resulting from hybridization of a double-stranded RNA precursor, although RNA folding algorithms did not reveal any significant secondary structure at, or in the vicinity of *Tg*-rdsRNA sequence matches (Figure 5C). Nonetheless, the detection of identical rdsRNA species in the related apicomplexan *N. caninum* and their isotype-specific accumulation (Figure 5B) together with their abundant loading into *Tg*-AGO (see below) make it unlikely that these species are simply random degradation products. This rather suggests the existence of a conserved mechanism that accounts for REP-dependent production of precursor molecules required for rdsRNA synthesis. A second class of low abundant *Tg*-rdsRNAs mapped directly to a long, imperfect stem-loop structure resulting from annealing of an individual ‘solo SDR’ unit. This structure is depicted in Figure 5D, together with the cloned sequences of contiguous or overlapping sRNAs that are likely produced via stepwise processing by the *Tg*-Dicer. Such imperfect structures might well represent the equivalent of the plant proto-*MIRNA* genes that arise from DNA-type non-autonomous elements known as miniature inverted-repeat transposable elements (MITEs). MITEs readily fold into imperfect stem-loops typical of miRNA precursors [53], [54] and often generate multiple sRNA species, including heterochromatic siRNA that dampen MITE expression transcriptionally, as well as recently-evolved (or young) miRNAs that may not have yet undergone positive selection for host transcript targeting, and tend to accumulate at low levels, as seen here with the SDR-derived *Tg*-rdsRNAs.

**Figure 6:**
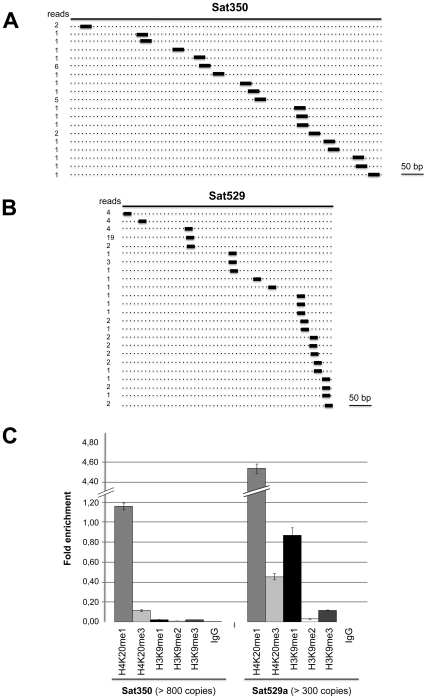
Satellite-associated *Tg*-satRNA localized to heterochromatin in *Toxoplasma*. New cloned *Tg*-satRNA species were assigned to repetitive satellite elements Sat350 and Sat529a, which are embedded in heterochromatin domains. *Tg*-satRNAs with perfect matches were plotted to satellite elements Sat350 (A) and Sat529a (B). The short thin lines below the long bars represent small RNAs derived from the sense strands with the reads indicated at the left. (C) Analyses of selected modified histones ratio at satellite Sat350 and Sat529a by quantitative chromatin immunoprecipitation PCR. Ratios of DNA precipitated with target modifications over DNA precipitated with core histone H3 were used to calculate relative precipitated fold enrichment shown on the *y-*axis. Experiments were triplicated and the data sets are concordant. Relative intensity is shown with standard deviations.

Sequence analysis also revealed the existence of a third class of repeat-associated sRNAs in *Toxoplasma*, which map perfectly to high-copy-number (>800 copies per genome) satellite DNA Sat350 (ABGTg/TGR family, Figure 6A) and Sat529a (Figure 6B) [55]. Although these satellite-associated (*Tg*-sat)RNAs had very low read numbers, they formed near-contiguous stretches of sequence along the corresponding *SAT* loci. These two features (low read-number, accumulation as populations rather than discrete species) are highly reminiscent of plant heterochromatic siRNAs found at DNA repeats and transposon loci with no intrinsic potential to form fold-back structures. In *Arabidopsis*, heterochromatic siRNAs are typically synthesized thought the conversion of aberrant RNA molecules into long-dsRNA, via the action of RDR2 [56]. Upon its processing by DCL3, the resulting siRNA population engages into AGO4 or AGO6 to mediate cytosine methylation and histone modifications at the sites of its production, resulting in heterochromatin formation [57]. We speculate that, similarly, *Tg*-satRNAs originate from the action of *T*g-RDR using *SAT*-derived aberrant transcripts as templates, and contribute to maintain the heterochromatic state found at both *SAT350* and *SAT529*, which, indeed, are enriched in silent chromatin marks including H4K20 and/or H3K9 monomethylations, as assessed by chromatin immunoprecipitation (Figure 6C). Similarly these silencing marks are also poorly but clearly enriched at REP- and MITE-derived sRNA loci (data not shown). We acknowledge that ChIP experiments for histone modifications only provide merely correlative evidence for a functional link between heterochromatin formation/spread and small RNA in *Toxoplasma* although some of the *Tg*-AGO-associated factors also support this idea (see later in the text). Assessing the formal contribution of *Tg*-AGO in DNA-based heterochromatic processes will require further experiments.

### sRNA loading, sub-cellular localization and polysome association of *Tg*-AGO

**Figure 7:**
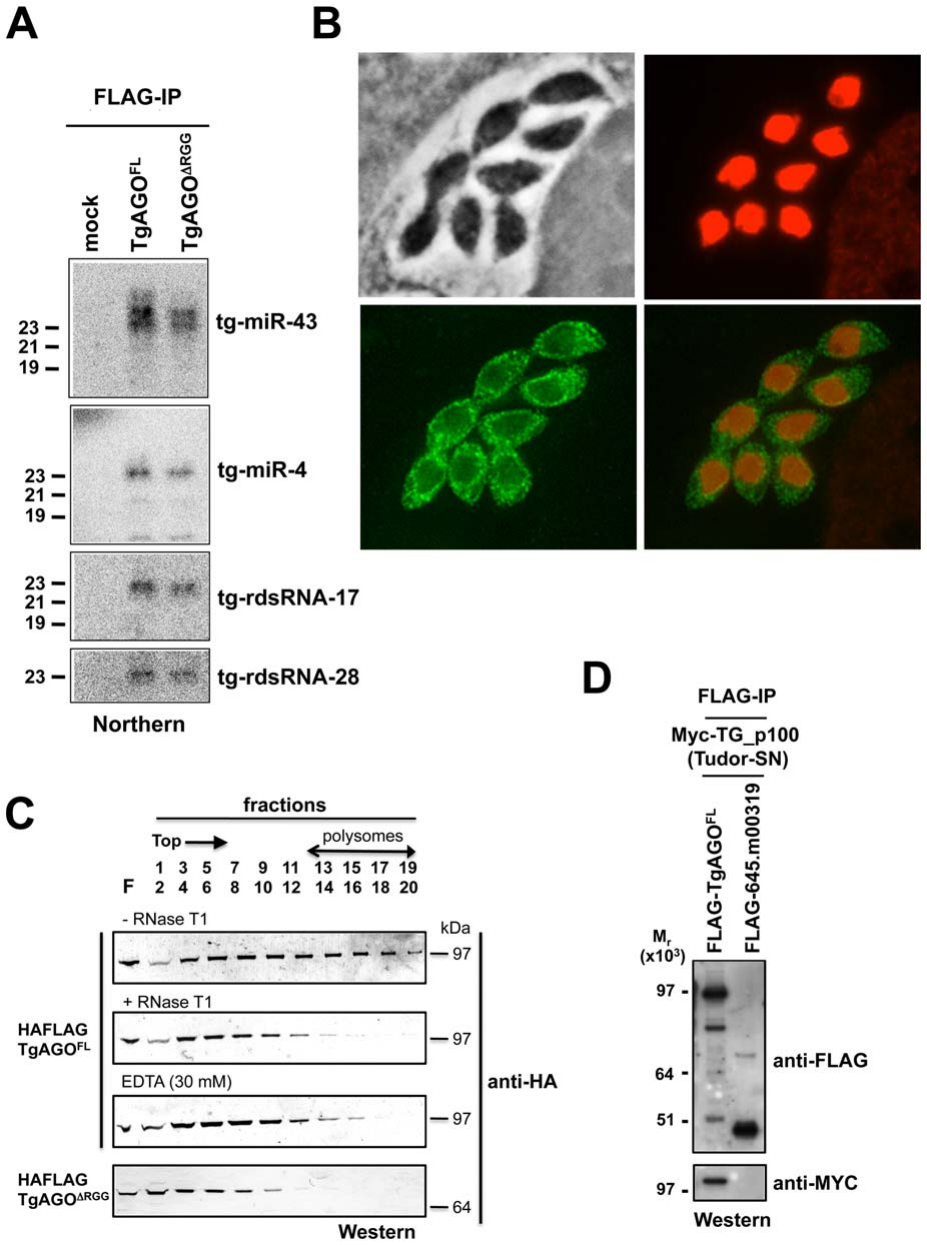
Small RNA loading, sub-cellular localization and polysome association of *Tg*-AGO. (A) Northern blot analyses of *Tg*-miRNAs and *Tg* -rdsRNAs associated with *Tg*-AGO. Immuno-precipitation using anti-Flag antibody was performed from RH strains expressing ectopically HAFlag-tagged either full-length or delta-RGG truncated *Tg*-AGO. RNAs isolated from the immunoprecipitates were probed for *Tg*-miR-4, *Tg*-miR-43, *Tg*-rdsRNA-17 and *Tg*-rdsRNA-28. Mock: non-immune IgG (negative control). (B) Stably expressed recombinant protein HAFlag-TgAGO^FL^ was detected by immunofluorescence assay using an HA antibody (in green) and compared to nuclear localization of acetylated histone H4 (in red). (C) To evaluate the sedimentation characteristics of *Tg-*AGO complexes, protein extracts containing HAFlag-TgAGO^FL^ or HAFlag-TgAGO^DRGG^ were subjected to sedimentation on 5%–45% sucrose gradients in the presence of cycloheximide (to preserve polyribosomes) or 30 mM EDTA (to disrupt polyribosomes). Aliquots of total extracts, either untreated (-RNase T1) or digested with RNase (+ RNase T1) were centrifuged through sucrose gradients and fractionated as described in Methods. Aliquots (equal volume) from indicated fractions were analyzed by Western blots with antibody against HA tag. (D) HAFlag-TgAGO^FL^ and HAFlag-645.m00319 (negative control) were transiently co-expressed with Myc-*Tg*-p100 (Tudor/SN). Following Flag affinity purification, the bound proteins were analyzed by Western blot using the anti-HA and the anti-myc antibodies. Molecular weight markers are indicated on the left.

In the absence of obvious, additional Ago-like proteins (including PIWI proteins) in the *Toxoplasma* genome (TOXODB, release v6.0), both transcriptional and post-transcriptional gene silencing events must, therefore, be operated via the same and unique *Tg-*AGO. To address this issue, we generated transgenic parasites expressing ectopically HAFlag-tagged, full-length *Tg-*AGO. RNP complexes were immuno-affinity purified (see next section), co-precipitated RNAs were extracted from the beads and analyzed by Northern using oligonucleotide probes specific to some of the highly abundant *Tg*-miRNAs and *Tg*-rdsRNAs studied above. *Tg*-miR-4 and -43, and as well *Tg*-rdsRNA-17 and -28 were indeed detected in the HaFlag-*Tg-*AGO immuno-precipitates but not in control immuno-precipitates (Figure 7A), indicating that *Tg*-AGO is a common effector of both types of sRNAs. This likely entails both cytoplasmic and nuclear distribution of the protein. Immunofluorescence and confocal microscopy revealed that *Tg-*AGO accumulates in tachyzoites mostly as granules of unidentified nature, but this labeling was superimposed over a diffuse cytoplasmic signal (Figure 7B and data not shown). Using acetylated histone H4 as a marker, confocal analyses also revealed a faint nuclear staining indicating that a minor portion of *Tg-*AGO localizes to the nucleus. Nuclear localization of *Tg-*AGO could be transient or highly dynamic, and under steady-state conditions. Alternatively, nuclear *Tg-*AGO could be incorporated into large protein complexes that prevent its optimal accessibility to antibodies.

The fact that a fraction of several *Tg*-miRNAs co-sediments with polysomes (Figure 4E, fractions 13–18) suggests that a portion of *Tg*-AGO should also be associated with polysomes, as has been shown for plant and metazoan miRNA-loaded AGOs [41], [58]–[62]. We thus examined the distribution of HaFlag-*Tg-*AGO using polysome gradients: cytoplasmic extracts from intracellular parasites were prepared and fractionated on sucrose gradients (Figure 7C). The absorbance profiles at 254 nm showed a pattern of ribosomes with ribosomal subunits, monosomes, and polysomes. Consistent with previous findings in metazoans [61], [62], most HAFlag-*Tg*-AGO was found near the top of the gradient, where soluble material and small ribonucleoprotein particles sediment. Some HAFlag-*Tg*-AGO was also heterodispersed throughout the gradient fractions, where polyribosomes and *Tg*-miRNAs co-sediment (Figures 4E and 7C). Treatments of cellular extracts with 30 mM EDTA or RNase T1, known to dissociate polysomes into ribosomal subunits and monosomes, caused a shift in HAFlag-*Tg*-AGO distribution from the denser fractions to the lighter fractions of the gradient (Figure 7C). This result suggests that a portion of miRNA-loaded *Tg*-AGO associates with polysomes to regulate translation of *Tg*-miRNA target mRNAs, perhaps in the cytoplasmic granules observed by immunofluorescence.

As noted previously, a characteristic feature of *Tg*-AGO is the presence, at the amino terminus, of a repeated RGG-rich region (amino acids 1–68), in which the arginine residues have the potential to undergo methylation (Figure 2B). This post-translational modification is known to influence the stability, activity and/or sub-cellular distribution of some metazoan AGO-like proteins [23]–[25]. Tudor-domain proteins specifically recognize symmetrically dimethylated arginines (sDMA) such as those found in AGO-like proteins [63], [64]. Accordingly, the immunopurified HAFlag-*Tg-*AGO complex (see below; Tables 1 and S4) was found to contain the Tudor-SN (tudor staphylococcal nuclease)/p100 homolog. Tudor-SN has five staphylococcal/micrococcal nuclease domains as well as Tudor domain, and it was described as a component of RISC in *Caenorhabditis elegans*, *Drosophila* and mammals [65]; more recently, the Tudor domain of the fly Tudor-SN was characterized as a specific sDMA-binding protein [64]. Reciprocal immunoprecipitation experiments further confirmed the specific binding of *Tg*-AGO to *Tg*-Tudor/SN (Figure 7D). Furthermore, HAFlag-*Tg-*AGO was also found to co-purify with *Tg*-PRMT1, which belongs to the family of arginine methyltransferases that use RGG motifs as substrates (Table 1). These results suggest that *Tg*-AGO is arginine-methylated, and that this modification might be specifically read by *Tg*-Tudor/SN, possibly to engage *Tg*-AGO into distinct modes of RNA silencing. In particular, the RGG-rich region of the *Trypanosoma brucei Tb*-AGO1 was found critical to its association with polysomes [66]. To test if the same was true of *Tg*-AGO, we engineered HAFlag-*Tg-*AGO^DRGG^, which carries a deletion of the RGG domain (amino acids 1 to 68). While HAFlag-*Tg-*AGO^DRGG^ was loaded normally with *T*g-miRNA and *T*g-rdsRNA (Figure 7A), the majority of the mutant protein was found near the top of polysome gradients, and was notably absent in fractions where polyribosomes sediment (Figure 7C). In addition, mass spectrometry analysis of the HAFlag-*Tg-*AGO^DRGG^ complex showed that it was no longer associated to *Tg*-Tudor/SN (data not shown). Thus, the proposed *Tg*-AGO arginine-methylation and association with *Tg*-Tudor/SN might allow post-loading sorting of distinct *Tg*-AGO-containing RNP complexes towards specific silencing modes. We note that its association to *Tg*-Tudor/SN through an RGG domain together with its predominant cytoplasmic localization evoke the as yet unexplored possibility that *Tg*-AGO may serve as a PIWI protein. Thus, in addition to its possible role in heterochromatin formation, *Tg-*AGO might contribute to post-transcriptional gene silencing of repeats and transposons via *Tg*-rdsRNAs, as is seen with metazoan PIWI proteins.

**Table 1:** Summary of *Tg*-AGO-associated proteins identified by mass spectrometry.

ToxoDB v4.3 ID	Band (peptides)	Homolog (H.s)	References
**RNA helicases**			
42.m00117	11 (18)	DDX3X/Bel (D.m)	40, 68
46.m00027	14 (9)	DDX17	39, 40
35.m00026	16 (20)	DDX39	39
583.m00676	15 (3)	DDX6/RCK	67
55.m04649	18 (5)	DDX48	39
46.m00023	12b (15)	FMR/FXR-like	39, 67
25.m02940	5 (9)	FMR/FXR-like	39, 67
**RNA binding proteins**			
49.m00009	20 (7)	p100 (Tudor/SN)	65
35.m00901	9a (53)	FUBP2/KSRP	40, 47
42.m03384	10a (20)	PABPC1	39, 40, 70
80.m02340	9a (17)	Nucleolin	39
57.m01690	15 (7)	HNRNPA3	40
46.m01699	15 (14)	HNRPH1	39, 40
55.m00241	21b (34)	HNRNPM	40
80.m00057	15 (5)	HNRNPL	39, 40
50.m00035	13a (4)	NOP56	39
**Translational machinery**			
20.m03912	9c (39)	EEF2	40
76.m00016	15 (42)	EEF1A1	40
42.m03276	21b (6)	EIF4E2	67
RPS and RPL	18–21c		40
**Transcription and Chromatin-related**			
TGME49_1053	4 (14)	TgCRC230	75
583.m00590	9c (5)	TBL1	75
42.m00014	16 (4)	HDAC1	75
583.m05395	3 (3)	AP2 transcription factor	
641.m01573	5 (4)	SMARCA2	
38.m00018	18 (4)	PRMT1	
55.m04747	8 (3)	RPB2	72
**Others**			
55.m00015	20 (25)	14.3.3 family	69, 71

### *Tg*-AGO-associated proteins constitute the near-entire cohort of previously identified human and fly miRNA-RISC components

**Figure 8:**
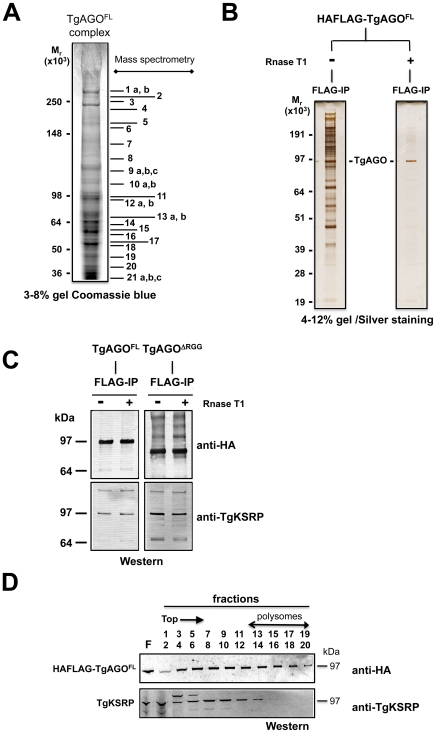
Proteomic analysis of *Tg*-AGO complexes. (A) *Tg*-AGO complexes were immunoprecipitated using Flag antibodies. The co-immunoprecipitated proteins were analysed using mass spectrometry (see Table 1 and Supplemental Table S1). Molecular weight markers are indicated on the left. (B) Transgenic parasites expressing ectopically HAFlag-TgAGO^FL^ were lysed and whole-cell-extracts prepared in the absence or presence (+RNase T1) of ribonuclease T1. HAFlag-TgAGO^FL^ was immunoprecipitated using anti-FLAG beads and flag peptide-eluted fractions were separated by SDS-polyacrylamide gel electrophoresis (4% to 12%), and visualized by silver staining. Molecular weight markers are indicated on the left. (C) Flag-immunopurified fractions from HAFlag-TgAGO^FL^ and HAFlag-TgAGO^DRGG^ RNase T1-treated and untreated samples were analyzed by Western blot using a home-made anti-*Tg*-KSRP antibody. (D) Sedimentation characteristics of *Tg*-KSRP compared to HAFlag-TgAGO^FL^.

To characterize the molecular composition of *Tg-*AGO-containing complexes, HAFlag-*Tg-*AGO was affinity purified from total cell extracts of intracellular tachyzoite by incubation with anti-FLAG agarose beads. The immunoprecipitated FLAG–protein complexes were eluted using the FLAG peptide. The immunoprecipitated proteins were then separated by SDS-PAGE, excised, and identified by mass spectrometry (Figure 8A). Tables 1 and S4 list the names of the identified proteins, which, remarkably, were direct orthologs of nearly all of the previously identified components of human and Drosophila miRNA-RISC [39], [40], [67]–[69]. These co-purified proteins fell within several functional groups. The largest group encompasses mRNA-binding proteins, in particular the heterologous nuclear ribonucleoproteins: HNRNPA3, HNRNPH1, HNRNPL, and HNRNPM [39], [40]. Several mRNA-binding proteins with putative functions in mRNA transport, stabilization and translation were also identified, including homologs of FUBP2/KSRP, nucleolin and FXR-related proteins, which are well known human and Drosophila Argonaute interactors [39], [40], [68]. Among the DEAD/DEAH box helicases, we found DDX17/DDX5, an ortholog of Drosophila p68, which has been shown to associate with Drosophila Ago2 [9], and DDX3X/Belle or DDX6/p54, which are all required for miRNA function (Table 1) [67], [68]. Consistent with the hypothesis that *Tg-*AGO associates with mRNPs, a homolog of the polyadenylation binding protein PABPC [70] was identified in the immuno-precipitate, indicating that mRNAs were present in the purifications. Accordingly, treatment of the lysate with RNase T1 prior to immuno-precipitation abolished the integrity of the *Tg*-AGO1 complex, indicating that the interactions between HAFlag-*Tg*-AGO and several proteins were RNA-mediated (Figure 8B). Identification of translation initiation and elongation factors, together with various 40S and 60S ribosomal proteins (Table S4) provides further support to the idea that the miRNA-loaded *Tg*-AGO, which associates with polysomes, might prevent translation of target mRNAs. Another noticeable partner of *Tg*-AGO was a ortholog of human FUBP2, also known as KHSRP/KSRP, which binds with high affinity to the terminal loop of some miRNA precursors and promotes their maturation [47]. *Tg*-KSRP might account, at least party, for the post-transcriptional regulation of some *Toxoplasma MIRNA* genes, as uncovered in this study (Figure 4B and 4C). Consistent with an effect of *Tg*-KSRP on miRNA maturation rather than activity, *Tg*-KSRP was found to bind *Tg*-AGO in an RNA-independent manner (Figure 8C). Accordingly, *Tg*-KSRP did not associate to polyribosomes (Figure 8D) and was also found in HAFlag-*Tg-*AGO^DRGG^ immuno-precipitates (Figure 8C).

Additional immuno-purified proteins are not obviously related to translational control but have been previously implicated as RISC-associated factors including *Tg*-Tudor/SN and *Tg*-PRMT1, already evoked above. Notably, *Tg*-AGO also co-purified with a conserved 14-3-3 protein (Table 1): 14-3-3 proteins that bind *S. pombe* Ago1 and human Ago2 are probably required for AGO protein functions in cell cycle and/or gene silencing pathways [71]. 14-3-3 proteins may also act as major regulators for the sorting of AGOs between distinct classes of RNA granules [69], which may include the *Tg-*AGO foci detected by immunofluorescence in the present study (Figure 7B). Collectively, these results provide compelling evidence that *Tg-*AGO is part of a functional RISC whose core components are nearly all orthologous to factors required for post-transcriptional gene silencing and its regulation in metazoans.

Consistent with additional, DNA-level silencing functions of *Tg*-AGO, the second largest subunit of *Tg-*RNA polymerase II (Rpb2) also co-purified in the HAFlag-*Tg-*AGO^FL^ immuno-precipitates (Table 1). Interestingly, a mutation of Rpb2 in fission yeast, *rpb2-m203*, disrupts coupling between transcription and siRNA processing in RNAi-dependent heterochromatin formation [72]. Also reminiscent of the fission yeast heterochromatic RNAi pathway, HAFlag-*Tg-*AGO was associated with the histone deacetylase TgHDAC3, a protein that may play similar roles to *S. pombe* Clr3 in the spread of heterochromatin [73], [74]. Remarkably, HAFlag-*Tg-*AGO was associated with all known components of the major transcriptional co-repressor complex *Tg*-CRC [75], [76], which contains the two repressor proteins *Tg*-CRC230 and *Tg*-TBL1, the catalytic subunit *Tg*-HDAC3 and a new plant-like AP2-domain transcription factor (Table 1). Moreover, peptide sequencing by tandem mass spectrometry indicated that the subunits of the complex are sub-stoichiometrically represented. This finding is consistent with the as yet unconfirmed idea that *Tg*-AGO-bound rdsRNA and possibly satRNAs may guide transcriptional gene silencing processes by recruiting histone deacetylases, and subsequently histone methylases (i.e. *Tg*-SET8 and *Tg*-SET3, [77]), to heterochromatic regions of the genome.

### Conclusions

The present analysis thus uncovers an unsuspected level of complexity in the RNA silencing pathways of the single cell parasite *T. gondii.* This complexity not only lies in the mere diversity of the sRNAs identified, but also in the apparent mix-and-matched nature of the silencing components found in this organism, both in terms of their evolution and function. In this respect, the *T. gondii* RNA silencing machinery and its usage by the parasite bewilder many accepted notions in the field. For instance, in no organism studied so far has a single Ago protein evolved to mediate both repeat-associated and miRNA-mediated gene silencing, two pathways usually considered drastically different. Likewise, the metazoan-like *Tg*-miRNAs have readily identifiable mRNA targets displaying perfect to near-perfect complementarity in both CDS and UTRs, which is unprecedented in animals. Further studies of the *Toxoplasma* RNA silencing pathways will undoubtedly reveal other surprises and, more importantly, might shed light on the molecular bases of virulence in this important Human parasite.

## Materials and Methods

### Parasite strains, cell culture, and *in vitro* bradyzoite differentiation

The parasite strains used in this study are the following: the *T. gondii* type I RH strain that has lost the ability to complete the two-host life cycle, the *T. gondii* type II Prugniaud strain that is capable of robust bradyzoite differentiation, and the *T. gondii* type III CTG and C56 strains. All *T. gondii* and *N. caninum* strains were maintained by serial passage in HFF monolayer under tachyzoite conditions in DMEM (Invitrogen) supplemented with 10% (vol/vol) FBS (Invitrogen). *T. gondii* type II Prugniaud strain was maintained under tachyzoite conditions in DMEM supplemented with 10% (vol/vol) FBS and 25 mM Hepes buffer, pH 7.2. To induce *in vitro* bradyzoite differentiation, extracellular tachyzoites were allowed to invade HFF cells for 16 hours, and the culture medium was removed and replaced by RPMI-1640 supplemented with 1% FBS and 50 mM Hepes buffer, pH 8.2. After 2–3 days of culture in alkaline medium, bradyzoite induction was assessed for P36 and SUMO expression by IFA as described previously [78]. The RHhxgprt- strain used in these studies contains a deleted or defective *HXGPRT* gene, which allows for the selection of transfected tachyzoites using mycophenolic acid.

### Antibodies

Antisera against *Tg*-FUBP2/KSRP (*35.m00901* gene) were produced by Eurogentec using the ‘*Super Speedy immunization*’ protocol and the following peptides *Tg*-FUBP2-1 (H2N-MARKKRGSAATPEEGC-CONH2) and *Tg*-FUBP2-2 (H2N-GTDKREDRGVTPEE DC-CONH2). Specific antibodies were affinity purified against both peptides. For immunoblot analysis purified antibodies were used at 1∶1000 dilutions. Primary antibodies for IFA, ChIP and Western blot included antibodies against haemagglutinin epitope tag (HA, Roche Diagnostic, dilution at 1∶1000), Polyclonal anti-H4-K20-1me (Abcam ab9051), Polyclonal anti-H4-K20-3me (Abcam ab9053), Polyclonal anti-H4-K20-1me (gift from Rice JC,Sims et al., 2006), Polyclonal anti-H4 Acetylated (K5-K8-K12-K16) (upstate 06-866), Anti-H3-K9-1me (upstate 07-450), Anti-H3-K9-2me (upstate 07-441), Anti-H3-K9-3me (upstate 07-442), Monoclonal anti-Myc (9E10 - sc40X, Santa-Cruz Bio.).

### Immunofluorescence microscopy

Infected HFFs grown on coverslips were washed in PBS and fixed/permeabilized for 20 min at room temperature with PBS containing 3% (vol/vol) formaldehyde and 0.2% Triton X-100 (vol/vol). Blocking was performed with PBS containing 5% FBS and 5% goat serum for 1 h at room temperature. Samples were incubated in PBS containing 1% FBS with the primary antibodies, followed by the secondary antibodies goat anti–mouse IgG coupled with Alexa Fluor 488 and goat anti–rabbit IgG coupled with Alexa Fluor 568 (Invitrogen) at a 1∶1,000 dilution each in PBS–1% FBS. Nuclei of host cells and parasites were stained for 10 min at room temperature with Hoechst 33258 at 2 µg/ml in PBS. After four washes in PBS, coverslips were mounted on a glass slide with Mowiol mounting medium (48 mM Tris-HCl [pH 8.5], 4.8% Mowiol 4–88 [wt/vol], 12% glycerol [vol/vol]), and images were acquired with a fluorescence microscope (Axioplan 2; Carl Zeiss, Inc.).

### Quantitative chromatin immuno-precipitation (QChIP) assay

QChIP assays were performed based on a modification of previously published methods [77], [79]. Immuno-precipitated DNA were purified through PCR Purification Kit columns (QIAGEN) and used as a template in semiquantitative QPCRs to detect specific targets. Specific primer pairs (melting temperature, 55 to 65°C) amplifying 200- to 450-bp fragments were used (supplemental Table S1). PCR was performed with 1 µL of DNA and 500 nM primers diluted to a final volume of 20 µL in SYBR Green Reaction Mix (Roche). Accumulation of fluorescent products was monitored by real-time PCR using a LightCycler 2.0 (Roche). Each PCR reaction generated only the expected specific amplicon, as shown by the melting-temperature profiles of final products (dissociation curve, automatically measured by the LightCycler 2.0) and by gel electrophoresis of test PCR reactions. No PCR products were observed in the absence of template. The fold difference of a given target sequence precipitated by a specific antibody was determined by dividing the amount of target sequence in the immunoprecipitate fraction by the amount of target sequence in input DNA (S8, S13). Real-time PCR was carried out in triplicate on 2 ng of DNA at 50°C for 2 min and 95°C for 10 min, followed by 40 cycles of 95°C for 15 s and 60°C for 1 min. Data were collected at 60°C. The concentration of primers and Taqman probes used was determined by following the optimization procedure described in PE Applied Biosystem's protocol. For each experiment, the threshold was set to cross a point at which real-time PCR amplification was linear. For the majority of the experiments, data were analyzed with a threshold of 0.05. Data collected was analyzed and plotted using Microsoft Excel.

Satellite 350B: OL17 (CGACTCGGACGTCAGGCCATGCAGAG) and OL18 (GCGCCTGAACAATACGCCCAACC).

Satellite 529A: OL19 (CTGCAGGGAGGAAGACGAAAGTTG) and OL20 (CTGCAGACACAGTGCATCTGGATT).

### Chromatographic purification of *Tg*-AGO-containing complexes

Whole-cell extract (WCE) from transgenic intracellular tachyzoites expressing ectopically HAFlag-*Tg*AGO^FL^ and HAFlag-*Tg*AGO^DRGG^ was incubated with 500 ml of anti-FLAG M2 affinity gel (Sigma) for 1 h at 4°C. Beads were washed with 10 column volumes of BC500 buffer [20 mM Tris (pH 8), 0.5 M KCl, 10% glycerol, 1 mM EDTA, 1 mM DTT, 0.1% NP40, 0.5 mM PMSF, aprotinin, leupeptide, pepstatin, 1 ug ml^−1^ each]. Bound peptides were eluted stepwise with 250 ug ml^−1^ FLAG peptide (Sigma) diluted in BC500 buffer. Each preparation was sufficiently clean such that individual peptide bands could be excised and sequenced by mass spectrometry.

### Mass spectrometry peptides sequencing

Protein bands were excised from colloidal blue-stained gels (Invitrogen), oxidized with 7% H_2_O_2_ and subjected to in-gel tryptic digestion. Peptides were extracted with 5% [v/v] formic acid solution and acetonitrile, and injected into an Ultimate 3000 (Dionex) nanoLC system that was directly coupled to a LTQ-Orbitrap mass spectrometer (Thermo Fisher Scientific). MS and MS/MS data were acquired using Xcalibur (Thermo Fischer Scientific) and processed automatically using Mascot Daemon software (Matrix Science). Tandem mass spectra were searched against a compiled *T. gondii* database using the MASCOT program (Matrix Sciences, London) available via intranet.

### Small RNA library preparation

RNA was extracted into TRIzol (Invitrogen), and aliquots of total RNA from RH strain were subjected to small RNA library construction as follows. To avoid any contamination with the host cell small RNAs, freshly released parasites were harvested from the culture supernatant, washed by centrifugation, and filtered through a 3-µm filter before use. For each library, 50 µg of total RNA was size fractionated on a 15% tris-borate-EDTA (TBE) urea polyacrylamide gel (Invitrogen) and a 19–40 base pair fraction was excised. RNA was eluted from the polyacrylamide gel slice in 300 µL of 0.3 M NaCl overnight at 4°C. The resulting gel slurry was passed through a Spin-X cellulose acetate filter column (Corning Inc.) and precipitated by the addition of 750 µL of ethanol and 3 µL of glycogen (5 mg/mL; Ambion). After washing with 75% ethanol, the pellets were allowed to air dry at 25°C and pooled in diethylpyrocarbonate (DEPC)-treated water. The 5′ RNA adapter (5′-GUUCAGAGUUCUACAGUCCGACGAUC-3′) was ligated to the RNA pool with T4 RNA ligase (Promega) in the presence of RNase Out (Invitrogen) 6 hours at 20°C. The ligation reaction was stopped by the addition of 2× Gel Loading Buffer II (Ambion). The ligated RNA was size fractionated on a Novex 15% TBE urea polyacrylamide gel (Invitrogen), and a 40–70 base pair fraction was excised. RNA was eluted from the polyacrylamide gel slice in 300 µL of 0.3 M NaCl overnight at 4°C. The RNA was eluted from the gel and precipitated as described above followed by resuspension in DEPC-treated water. The 3′ RNA adapter (5′-pUCGUAUGCCGUCUUCUGCUUGUidT-3′; p, phosphate; idT, inverted deoxythymidine) was subsequently ligated to the precipitated RNA with T4 RNA ligase (Ambion) in the presence of RNase Out (Invitrogen) 6 hours at 20°C. The ligation reaction was stopped by the addition of 2× Gel Loading Buffer II (Ambion). Ligated RNA was size fractionated on a Novex 10% TBE urea polyacrylamide gel (Invitrogen), and the 70–100 base pair fraction was excised. The RNA was eluted from the polyacrylamide gel and precipitated from the gel as described above and resuspended in 4.5 µL of DEPC-treated water. The RNA was converted to single-stranded cDNA using Superscript II reverse transcriptase (Invitrogen) and Illumina's small RNA RT-Primer (5′-CAAGCAGAAGACGGCATACGA-3′) following the manufacturer's instructions. The resulting cDNA was PCR-amplified with Phusion™ High Fidelity DNA Polymerase (NEB) in 15 cycles using Illumina's small RNA primer set (5′-CAAGCAGAAGACGGCATACGA-3′; 5′- AATGATACGGCGACCACCGACAGGTTCAGAGTTCTACAGTCCGA-3′). PCR products were purified on a Novex 6% TBE PAGE gel (Invitrogen) and the 100 base pair fraction was excised. The DNA was eluted into 100 µL of 1x NEBuffer 2 at room temperature for 2 hours. The resulting gel slurry was passed through a Spin-X filter (Corning) and precipitated by the addition of 325 µL of ethanol, 10 µL of 3 M sodium acetate, and 3 µL of glycogen (5 mg/mL; Ambion). After washing with 75% ethanol, the pellet was allowed to air dry at 25°C and dissolved in 10 µL of resuspension buffer (10 mM Tris-HCl, pH 8.5). The purified PCR products were quantified on the Agilent DNA 1000 chip and diluted to 10 nM for sequencing on the Illumina 1G (GATC BIOTECH, Konstanz, Germany).

### Northerns

RNA from *Toxoplasma* and *Neospora* strains was extracted into TRIzol (Invitrogen), deproteinized with phenol chloroform/isoamyl alcohol, and RNA was recovered by ethanol precipitation. For small RNA analyses, 30 ug of purified RNA were separated on a 15% polyacrylamide (w/v) 8 M urea gel and transferred to GeneScreen nylon membranes. DNA oligonucleotides complementary to tg-miRNAs or tg-rasiRNAs were labeled with [g-32P]ATP using T4 PNK (Promega). Hybridizations were performed at 37°C overnight. Hybridized membranes were exposed to imaging plates that were recorded after 5 h (PhosphoImager, FLA-8000, *Fuji*).

### Polyribosome analysis

Whole cell extracts were prepared as described previously [59] but using a modified polysome buffer containing 100 mM NaCl, 40 mM Tris-Hcl pH 7, 10 mM MgCl2, 1 mM DTT, 1% Triton TX100 and protease inhibitor (Complete). Cycloheximide (100 ug/ml) was added to cells (10 min at 37°C) prior to collecting the cells by centrifugation. The drug was present in all buffers throughout the entire procedure. For polysome fractionation experiment, approximately 1000 OD_600_ of whole cell extract were layered onto 12 ml 5%–40% sucrose gradients prepared in polysome buffer without Triton, and centrifuged at 4°C for 2h at 36,000 rpm in a Beckman SW41 rotor. After centrifugation, 500 ml fractions were collected from the top of the gradient and the 260-nm absorbance profile was recorded. For Northern-Blot analysis, RNAs from each fraction were precipitated by adding 0,1M NaCl and 3 volumes ethanol and extracted with the TRIzol method. For Western-Blot analysis, 15 ul of each gradient fraction were run on 12% SDS-PAGE gels.

### b-Elimination reaction of small RNAs

Total RNAs (30 ug) from RH-infected fibroblasts were incubated in a solution containing 10 mM HEPES (pH 7.0) and 250 mM sodium periodate for 30 min at 22°C. An equal volume of formamide loading dye was added to the samples, followed by incubation for 45 min at 99°C. The reaction mixture was then analysed by Northern-Blot. An equal amount of untreated RNA was also loaded onto the gel for comparison. RNA oligos of known sequence (19, 21 and 23 nt synthetic RNA oligos) were also treated with sodium periodate to check for the completion of b-elimination reaction, and the blots were probed with end-labeled oligos complementary to the synthetic oligos.

### Computational analyses

We analysed a pool of 5,701,506 raw reads obtained by the sequencing-by-synthesis (*Illumina*). Initially, all the sequences fully matching tRNAs or rRNAs were removed. The remaining sequences were used to build a local Mysql database and then trimmed in a 4 steps process: 1) We removed 3′ and 5′ adaptor sequences, using an iterative scheme and updated the database. 2) We removed from each sequence the nucleotides, in the 5′ and 3′ extremities, with a Phred Quality Score (http://www.phrap.com/phred/) below 10 and updated the database. 3) We eliminated all the reads containing more than 6 stretches of C, T or G. 4) We screened the database to keep only reads having a length >19 nucleotides and an average Phred Quality Score >15. The final pool of 1,555,290 reads was used to cluster small RNAs. The sequences without any variation were classified in the same cluster. A total of 275,888 distinct clusters were identified and used for further analysis. All these clusters were compared against the *T. gondii* genome (http://www.ToxoDB.org, version 4.3) using Blast program with specific parameters (Word size set to 4 and Penalty for a nucleotide mismatch set to -1). This configuration allowed a more refined search of small versus large nucleotide sequences. All the results were saved in the database and used for mapping sRNAs on the chromosomes. Based on the chromosome position, we classify the clusters into families. First, we seeded our classification with results having 100% identity and an alignment length >19 and then recovered the clusters varying with less than three nucleotides. All further analyses were focused on the most abundant families. Images, multi-fastas and alignments against *T. gondii* genome were automatically generated and manually curated. All these treatments were made using an in-house API (Genobrowser) and functionalities (tools unpublished) written in PHP.

### Identification of miRNA candidate loci

Up to 10 sequence windows on both strands, spanning the locus and including variable lengths of flanking regions (5–200 bp on either side), were examined for their potential to form fold-back transcripts by using the RNAfold [80] and Mfold [81] programs. The predicted outcomes, including the minimal folding free energy (MFE), at least 20 kcal/mole (dG = −20 kcal/mole), the length of pre-miRNAs, and the number of nucleotides (A, C, G, or U) in each pre-miRNA were recorded and used for further analysis. Each predicted tg-microRNA was further checked manually to ensure that they were from good quality single-stranded hairpins and that a miRNA/miRNA* pair had 0–2 nt 3′ overhangs.

### Construction of *T. gondii* expression vector

The coding sequence of *Tg*-AGO (genbank, GU046561) was amplified by RT-PCR to introduce BamHI and HindIII sites at the start and the stop codons respectively. Primers forward (5′-ggatccATGAACGGAGGAGGCAGAGGAAGAG-3′) and reverse (5′- aagcttCCATCAATGCTGTCTCAACAGAAC-3′) were used for PCR amplification. The PCR product allowed the cloning of Tg-AGO^FL^ in frame with an N-terminal HAFlag tag into the *T. gondii* expression vector pMAH14 (GRA1 promoter, [75]) digest with BglII-HindIII. *Tg-*AGO^DRGG^ was amplified by PCR with forward (5′- ggatccCTGTACGATGGAGACCACCTTCTC-3′) and reverse (5′- aagcttCCATCAATGCTGTCTCAACAGAAC-3′) and cloned subsequently in pMAH14 (BglII-HindIII).

## Supporting Information

Figure S1 *T. gondii* small RNAs cloning. (A) Ethidium bromide was used to visualize small RNAs in total RNA extracted from *Toxoplasma* cultures. A spiked synthetic RNA oligonucleotides were used as a size reference. RNA markers (middle lane) are 19, 24 and 33 nucleotides. (B) Genome distribution of *Toxoplasma* small RNAs.
(1.20 MB TIF)

Figure S2 *Tg*-microRNA-60 family. (A) A miR-60b production spot on chromosome VIIa is depicted with the representative secondary structure of the precursor and the conservation across parasite species. Small RNAs with perfect matches were plotted within a 800-bp sliding window. Short thin lines above the long bars represent small RNAs derived from the antisense strands, and lines below the bars represent small RNAs from the sense strands. Vertical bars represent the consensus positions of sequencing reads that mapped to the predicted precursors and numbers indicate the total number of these reads. Fold-back structure of the precursor was predicted with mfold. The mature region is shown in red. Sequence conservation across the three canonical strains of *T. gondii* and *N. caninum* are shown. (B) *Toxoplasma* Tg-miR-60 family variants are aligned. Number of reads are indicated for each species. There is 3′ heterogeneity among the sequenced clones for most miRNAs.
(0.58 MB TIF)

Figure S3 *Tg*-microRNA-4 family. (A) A miR-4 production hot spot in chromosome V is shown along with the predicted structure and the sequence conservation across parasite species. Same legend as in Figure S2A. The mature region is shown in red. (B) *Toxoplasma Tg*-miR-4 family variants are aligned. The numbers of reads are indicated for each species. There is 3′ heterogeneity among the sequenced clones for most miRNAs.
(0.63 MB TIF)

Figure S4 Characteristics of *Tg*-miR-15 and -49. The miR-15/miR-49a (A) and miR49b (B) production hot spots in chromosome VIIb are shown alongside the predicted structure and the sequence conservation across parasite species. The mature region is shown in red for miR15 and miR49b and in green for miR-49a. Same legend as in Figure S2A.
(0.71 MB TIF)

Figure S5 Characteristics of the *Tg*-miR-40 family. The miR-40a (A) and miR40b (B) production hot spots in chromosomes X and VIIb respectively are shown along with the predicted structure and the sequence conservation across parasite species. The mature region is shown in red. A new cloned *Tg*-miR candidate is labelled in green. Same legend as in Figure S2A.
(0.97 MB TIF)

Figure S6 Characteristics of *Tg*-miR-43. (A) A miR-43 production hot spot in chromosome IX is shown along with the predicted structure (B) and the sequence conservation across parasite species (C). The mature region is shown in red. Same legend as in Figure S2A.
(0.44 MB TIF)

Figure S7 Characteristics of *Tg*-miR-56. (A) A miR-56 production hot spot in chromosome XI is shown along with the predicted structure (B) and the sequence conservation across parasite species (C). The mature region is shown in red. Same legend as in Figure S2A.
(0.42 MB TIF)

Figure S8 Characteristics of the *Tg*-miR-24 family. (A) miR-24a/miR-24b production hot spots are shown in chromosome VIII along with the predicted structure and (B) the sequence conservation across parasite species. The mature region is shown in red and the passenger strand (microRNA*) in blue. Same legend as in Figure S2A.
(0.95 MB TIF)

Figure S9 Characteristics of *Tg*-miR-66. (A) A miR-66 production hot spot in chromosome XII is shown along with the predicted structure and (B) the sequence conservation across parasite species. The mature region is shown in red and the passenger strand (microRNA*) in blue. Same legend as in Figure S2A.
(0.59 MB TIF)

Figure S10 Characteristics of *Tg*-miR-62. (A) A miR-62 production hot spot in chromosome IX is shown along with the predicted structure and (B) the sequence conservation across parasite species. The mature region is shown in red and the passenger strand (microRNA*) in blue. Same legend as in Figure S2A.
(0.46 MB TIF)

Figure S11 Characteristics of *Tg*-miR-64. (A) A miR-64 production hot spot in chromosome XI is shown along with the predicted structure and (B) the sequence conservation across parasite species. The mature region is shown in red. Same legend as in Figure S2A.
(0.52 MB TIF)

Figure S12 Characteristics of *Tg*-miR-65. (A) A miR-65 production hot spot in chromosome IX is shown along with the predicted structure and (B) the sequence conservation across parasite species. The mature region is shown in red and the passenger strand (microRNA*) in blue. Same legend as in Figure S2A.
(0.45 MB TIF)

Figure S13 Characteristics of *Tg*-miR-61 and -63. (A) Predicted structure of miR-61 is shown along with (B) the sequence conservation across parasite species. The pre-miR-61 stem-loop structure is conserved across three loci on chromosomes IX and Ia. (C) Predicted structure of miR-63 is shown along with (D) the sequence conservation across parasite species. The mature region is shown in red and the passenger strand (microRNA*) in blue. Same legend as in Figure S2A.
(0.79 MB TIF)

Figure S14 Northern analysis of *Tg*-miR and *Tg*-rdsRNAs. *Full-size* images of RNA blot phosphoimager scans used to generate panels A and B in Figures 4 and 5, respectively. Same legend as in Figures 2A and 3A. RNA markers (left lane) are 19, 21 and 23 nucleotides.
(2.37 MB TIF)

Figure S15 Identification of putative *Toxoplasma* proto-microRNAs. Secondary structure of *T. gondii* proto-miR-1 (A), -2 (B), -3 (C) and -4 (D) foldbacks compared to predicted secondary structure of the orthologous sequences from *N. caninum*. The red line indicates the cloned mature *T. gondii* miRNA sequence, while the blue line refers to the corresponding *N. caninum* sequence. Number of reads: *Tg*-proto-miR-1 (158 reads), *Tg*-proto-miR-2 (109 reads), *Tg*-proto-miR-3 (138 reads) and *Tg*-proto-miR-4 (107 reads).
(0.93 MB TIF)

Figure S16 Prediction of *Toxoplasma* miRNA target genes. (A) The number of predicted target genes are shown for 12 *Tg*-microRNAs. (B) Genes targeted by the 14 *Tg*-miRNA families were functionally classified using the eukaryotic Clusters of Orthologous Groups (KOG) database.
(1.07 MB TIF)

Table S1 *Toxoplasma* tg-microRNAs.
(0.03 MB PDF)

Table S2 Predicted binding sites of selected *T. gondii* tg-miRNA.
(0.04 MB PDF)

Table S3 *Toxoplasma* REP-derived small RNAs (rdsRNAs).
(0.03 MB PDF)

Table S4 Summary of *Tg*-AGO-associated proteins identified by mass spectrometry.
(0.12 MB PDF)
