# Supplementary figures and images for: A Complex Small RNA Repertoire Is Generated by a Plant/Fungal-Like Machinery and Effected by a Metazoan-Like Argonaute in the Single-Cell Human Parasite Toxoplasma gondii
Source: PLoS Pathog. 2010 May 27;6(5):e1000920. doi: 10.1371/journal.ppat.1000920 (PMC2877743; doi:10.1371/journal.ppat.1000920)

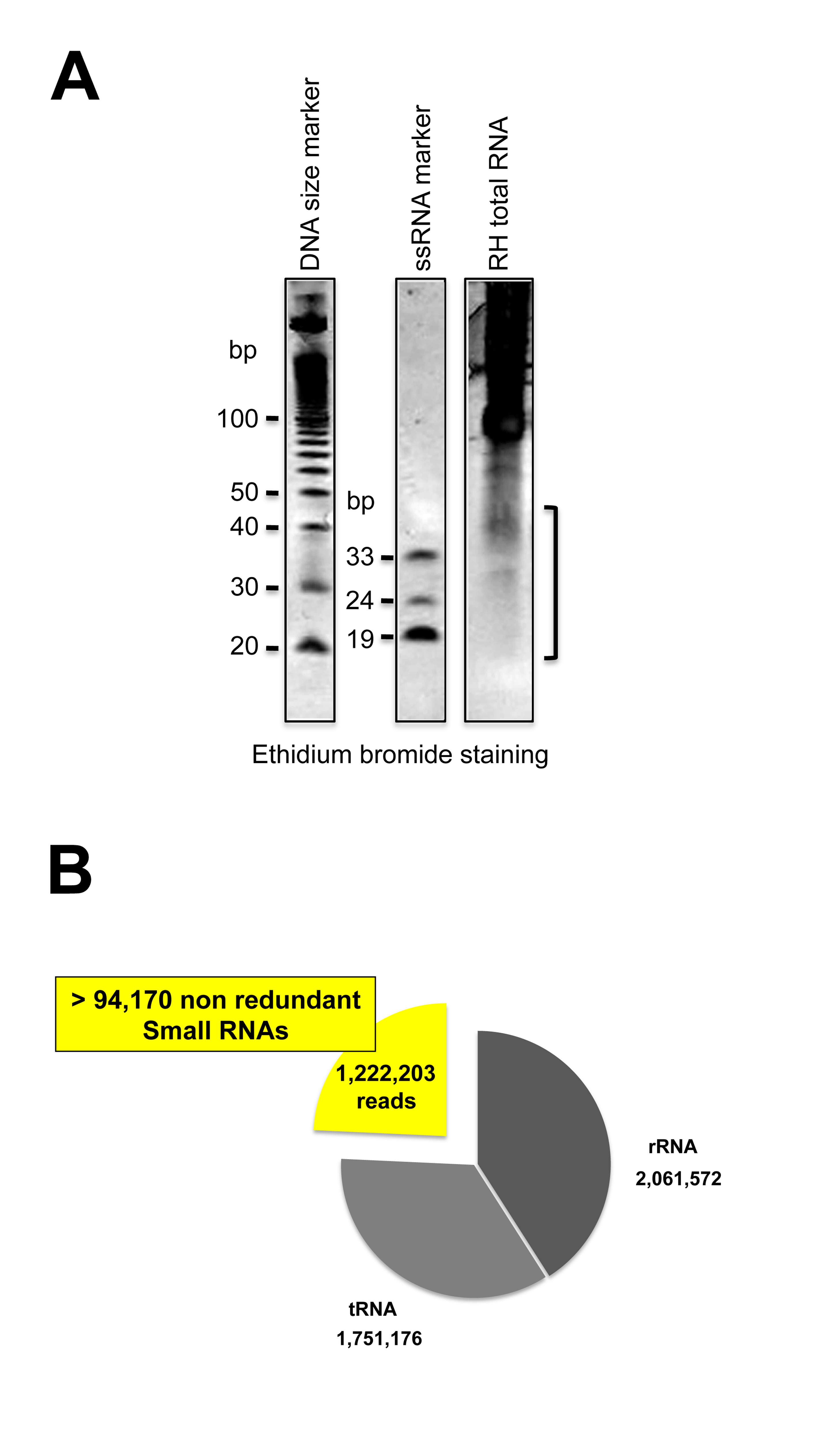

Supplement: Figure S1 — T. gondii small RNAs cloning. (A) Ethidium bromide was used to visualize small RNAs in total RNA extracted from Toxoplasma cultures. A spiked synthetic RNA oligonucleotides were used as a size reference. RNA markers (middle lane) are 19, 24 and 33 nucleotides. (B) Genome distribution of Toxoplasma small RNAs. (1.20 MB TIF) [file ppat.1000920.s001.tif]

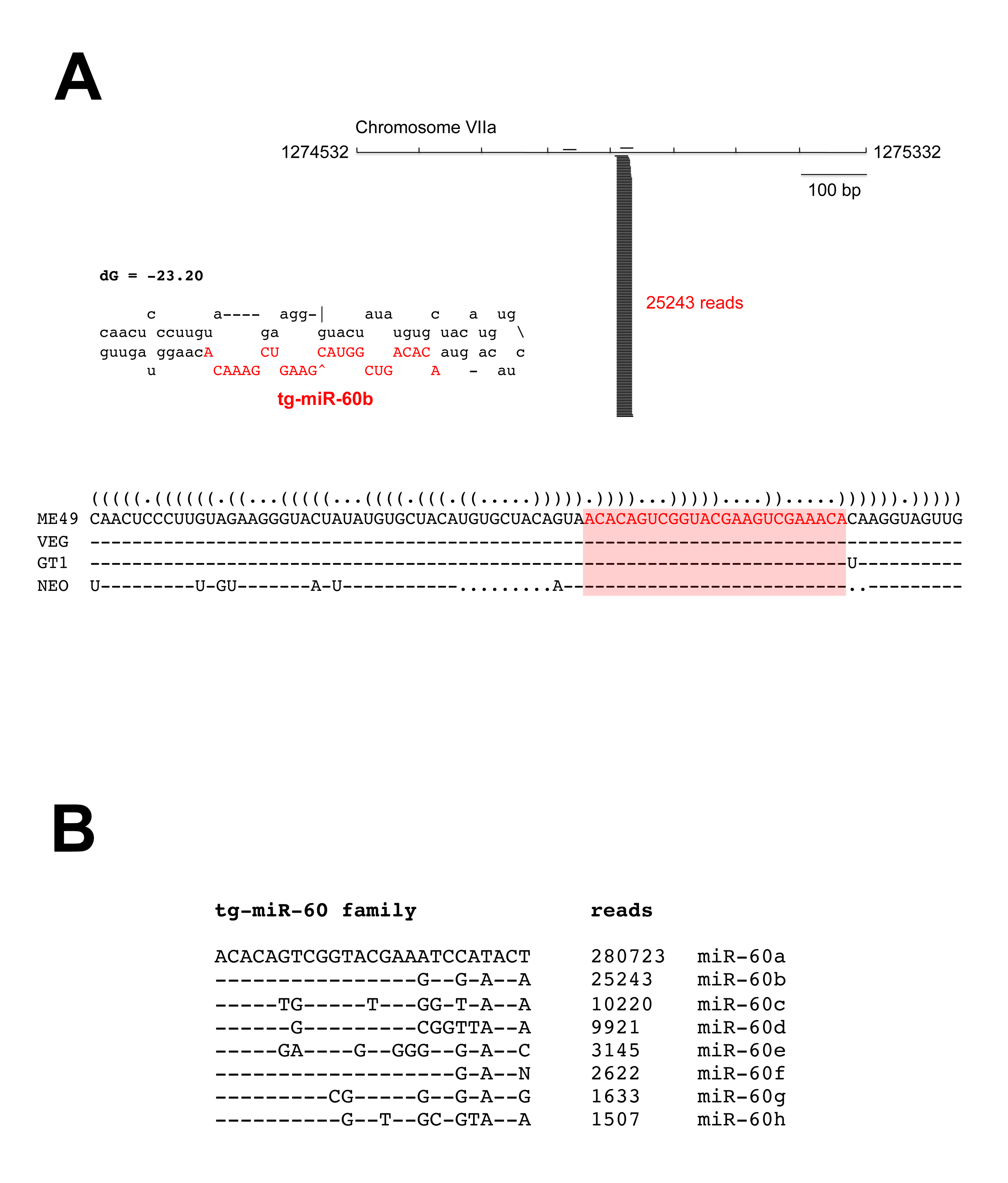

Supplement: Figure S2 — Tg-microRNA-60 family. (A) A miR-60b production spot on chromosome VIIa is depicted with the representative secondary structure of the precursor and the conservation across parasite species. Small RNAs with perfect matches were plotted within a 800-bp sliding window. Short thin lines above the long bars represent small RNAs derived from the antisense strands, and lines below the bars represent small RNAs from the sense strands. Vertical bars represent the consensus positions of sequencing reads that mapped to the predicted precursors and numbers indicate the total number of these reads. Fold-back structure of the precursor was predicted with mfold. The mature region is shown in red. Sequence conservation across the three canonical strains of T. gondii and N. caninum are shown. (B) Toxoplasma Tg-miR-60 family variants are aligned. Number of reads are indicated for each species. There is 3′ heterogeneity among the sequenced clones for most miRNAs. (0.58 MB TIF) [file ppat.1000920.s002.tif]

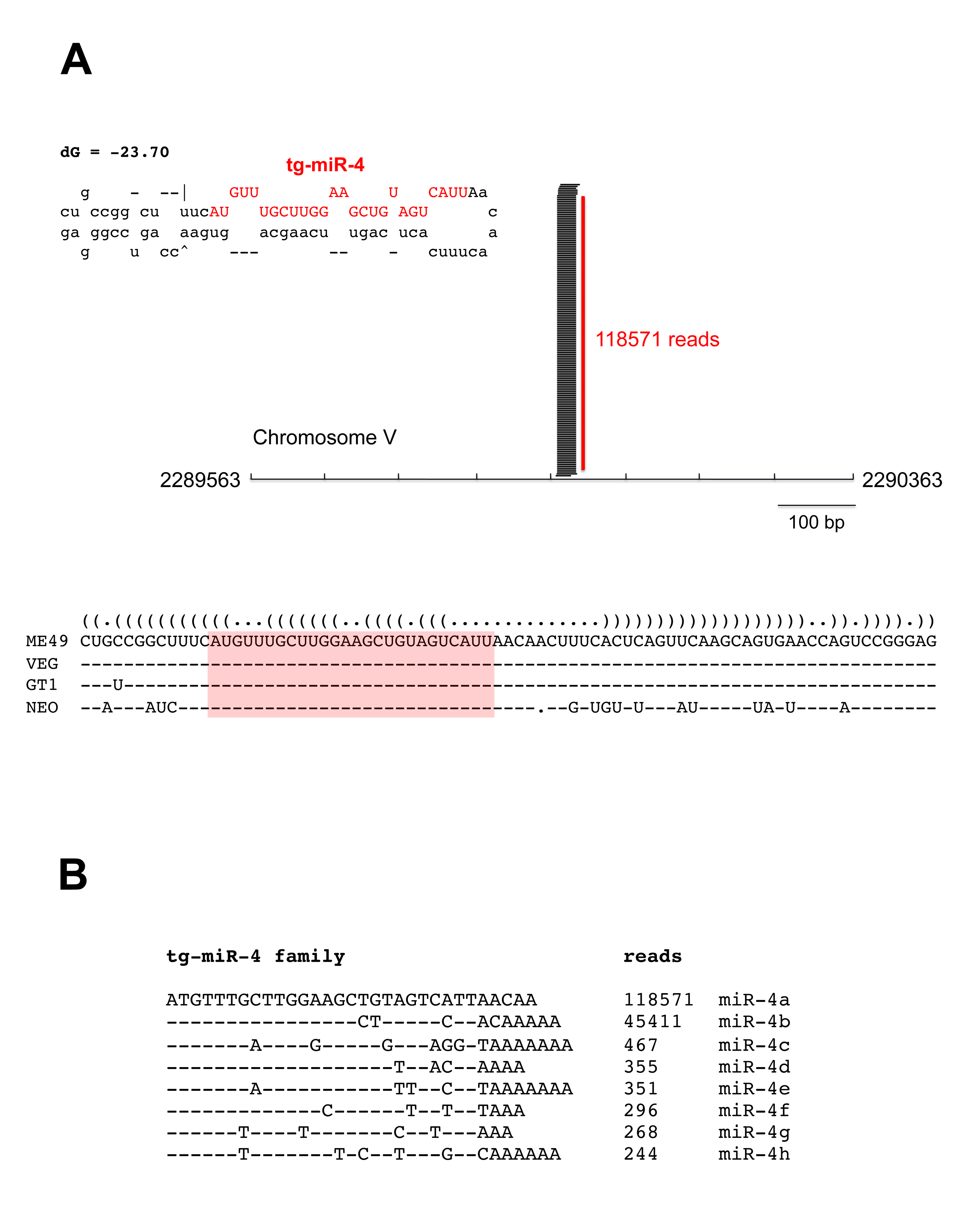

Supplement: Figure S3 — Tg-microRNA-4 family. (A) A miR-4 production hot spot in chromosome V is shown along with the predicted structure and the sequence conservation across parasite species. Same legend as in Figure S2A. The mature region is shown in red. (B) Toxoplasma Tg-miR-4 family variants are aligned. The numbers of reads are indicated for each species. There is 3′ heterogeneity among the sequenced clones for most miRNAs. (0.63 MB TIF) [file ppat.1000920.s003.tif]

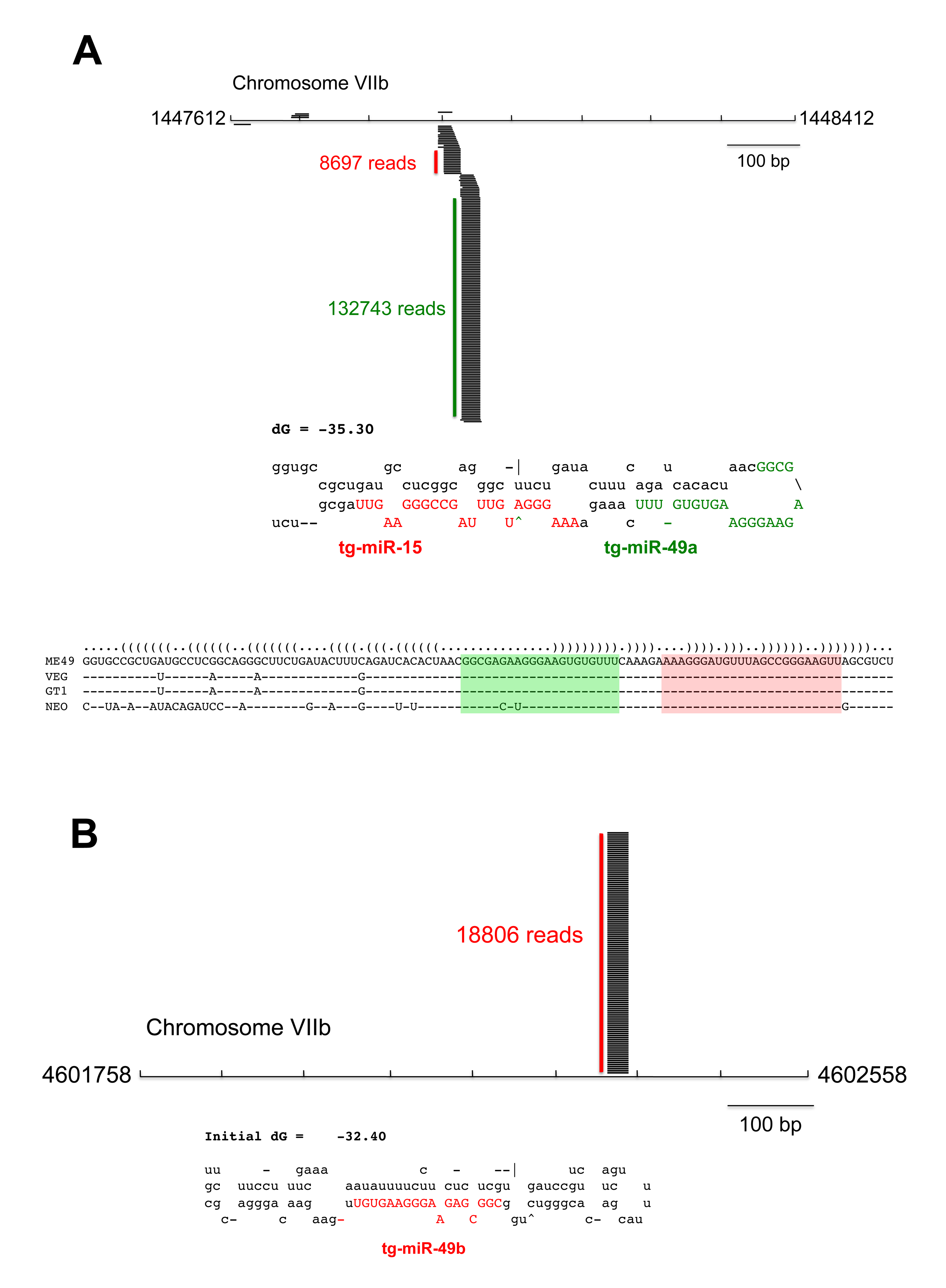

Supplement: Figure S4 — Characteristics of Tg-miR-15 and -49. The miR-15/miR-49a (A) and miR49b (B) production hot spots in chromosome VIIb are shown alongside the predicted structure and the sequence conservation across parasite species. The mature region is shown in red for miR15 and miR49b and in green for miR-49a. Same legend as in Figure S2A. (0.71 MB TIF) [file ppat.1000920.s004.tif]

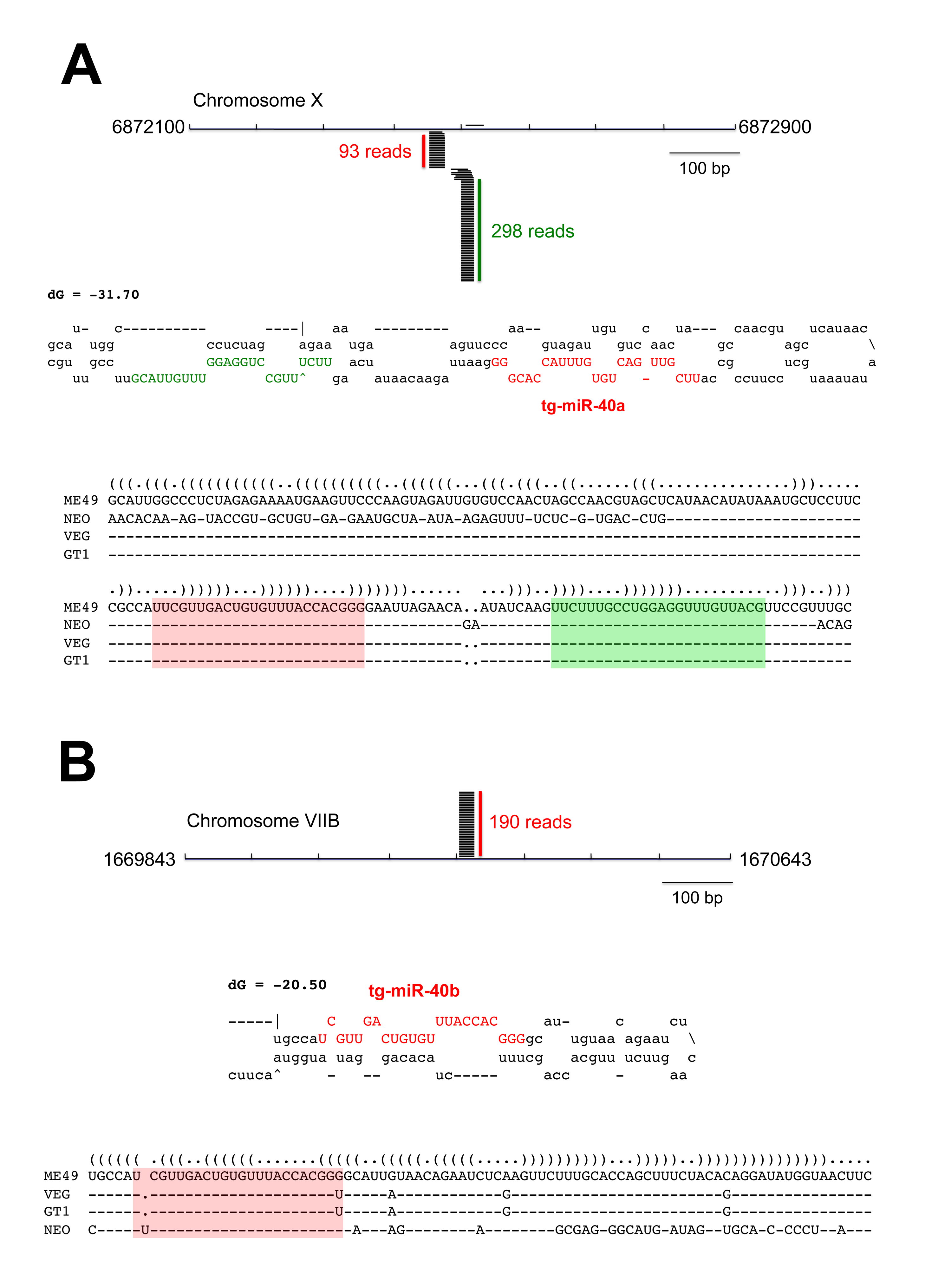

Supplement: Figure S5 — Characteristics of the Tg-miR-40 family. The miR-40a (A) and miR40b (B) production hot spots in chromosomes X and VIIb respectively are shown along with the predicted structure and the sequence conservation across parasite species. The mature region is shown in red. A new cloned Tg-miR candidate is labelled in green. Same legend as in Figure S2A. (0.97 MB TIF) [file ppat.1000920.s005.tif]

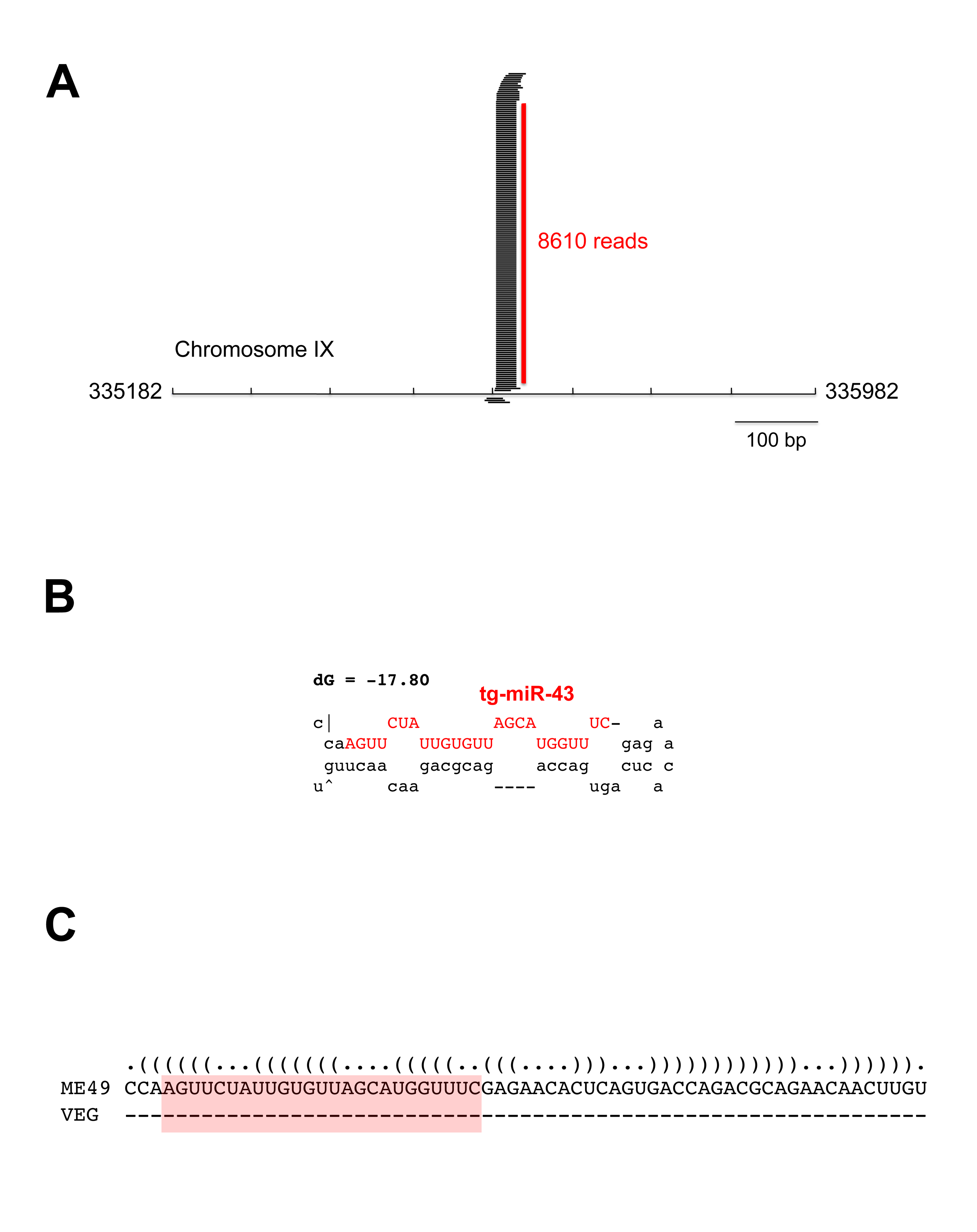

Supplement: Figure S6 — Characteristics of Tg-miR-43. (A) A miR-43 production hot spot in chromosome IX is shown along with the predicted structure (B) and the sequence conservation across parasite species (C). The mature region is shown in red. Same legend as in Figure S2A. (0.44 MB TIF) [file ppat.1000920.s006.tif]

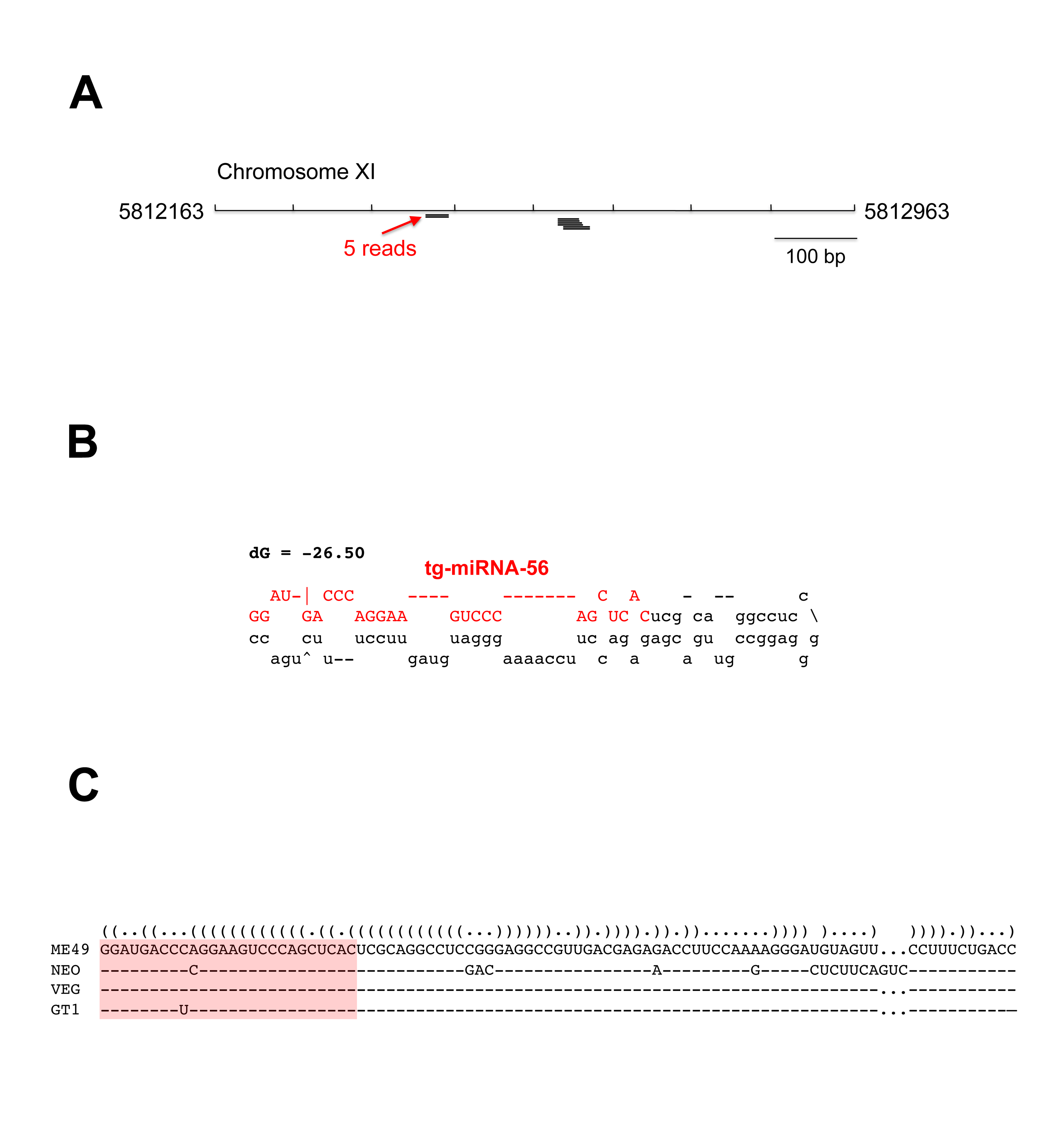

Supplement: Figure S7 — Characteristics of Tg-miR-56. (A) A miR-56 production hot spot in chromosome XI is shown along with the predicted structure (B) and the sequence conservation across parasite species (C). The mature region is shown in red. Same legend as in Figure S2A. (0.42 MB TIF) [file ppat.1000920.s007.tif]

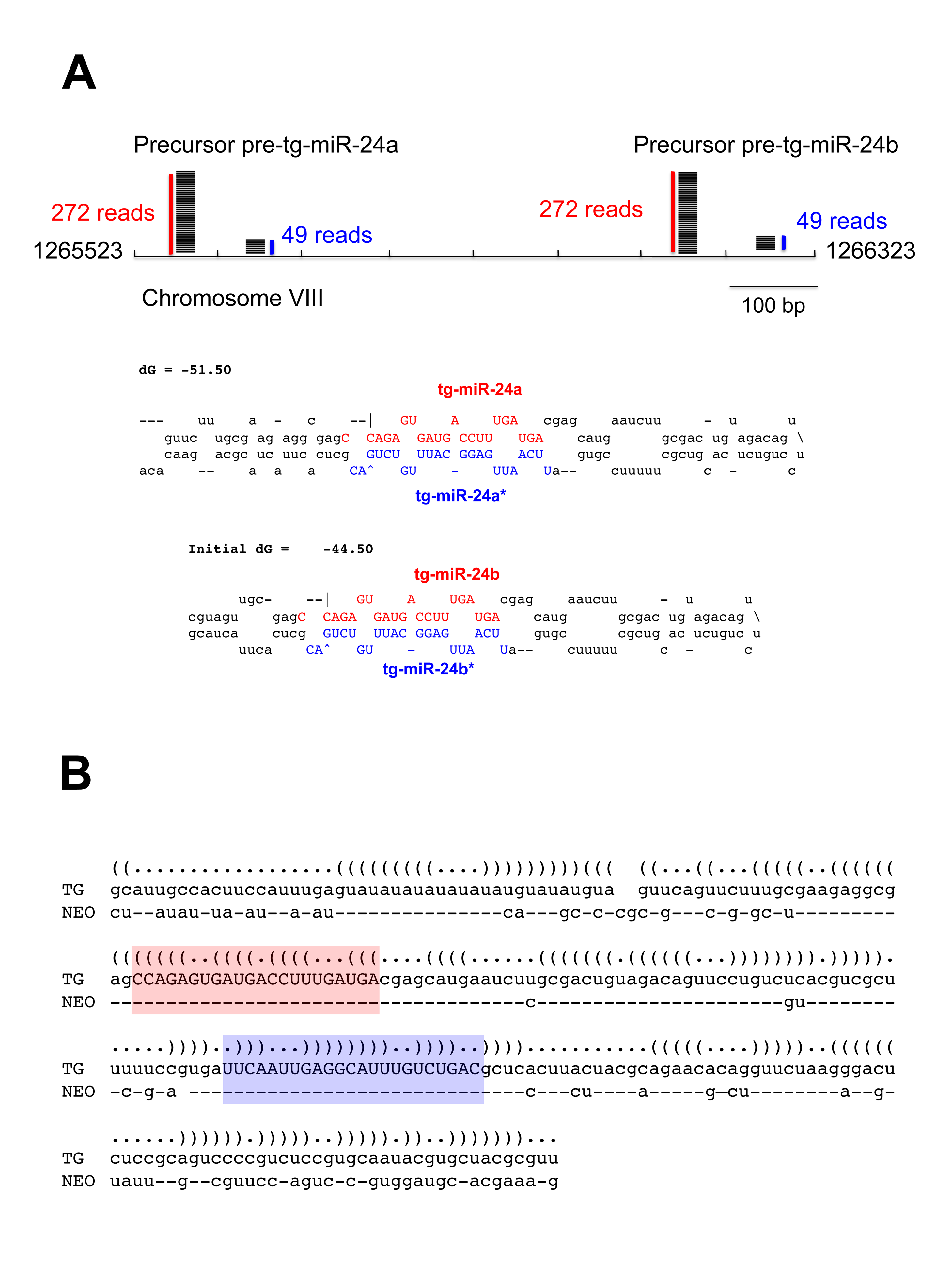

Supplement: Figure S8 — Characteristics of the Tg-miR-24 family. (A) miR-24a/miR-24b production hot spots are shown in chromosome VIII along with the predicted structure and (B) the sequence conservation across parasite species. The mature region is shown in red and the passenger strand (microRNA*) in blue. Same legend as in Figure S2A. (0.95 MB TIF) [file ppat.1000920.s008.tif]

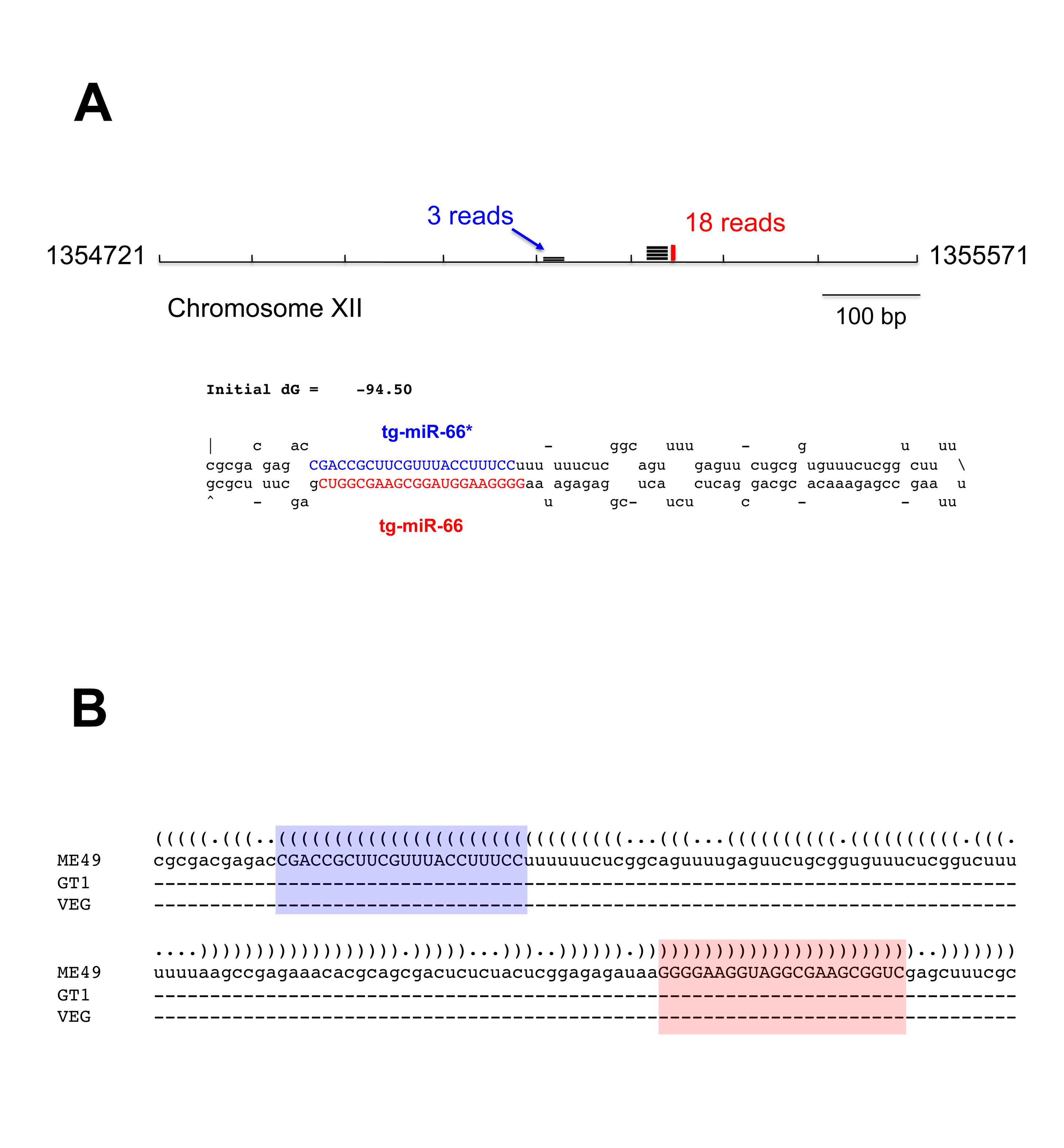

Supplement: Figure S9 — Characteristics of Tg-miR-66. (A) A miR-66 production hot spot in chromosome XII is shown along with the predicted structure and (B) the sequence conservation across parasite species. The mature region is shown in red and the passenger strand (microRNA*) in blue. Same legend as in Figure S2A. (0.59 MB TIF) [file ppat.1000920.s009.tif]

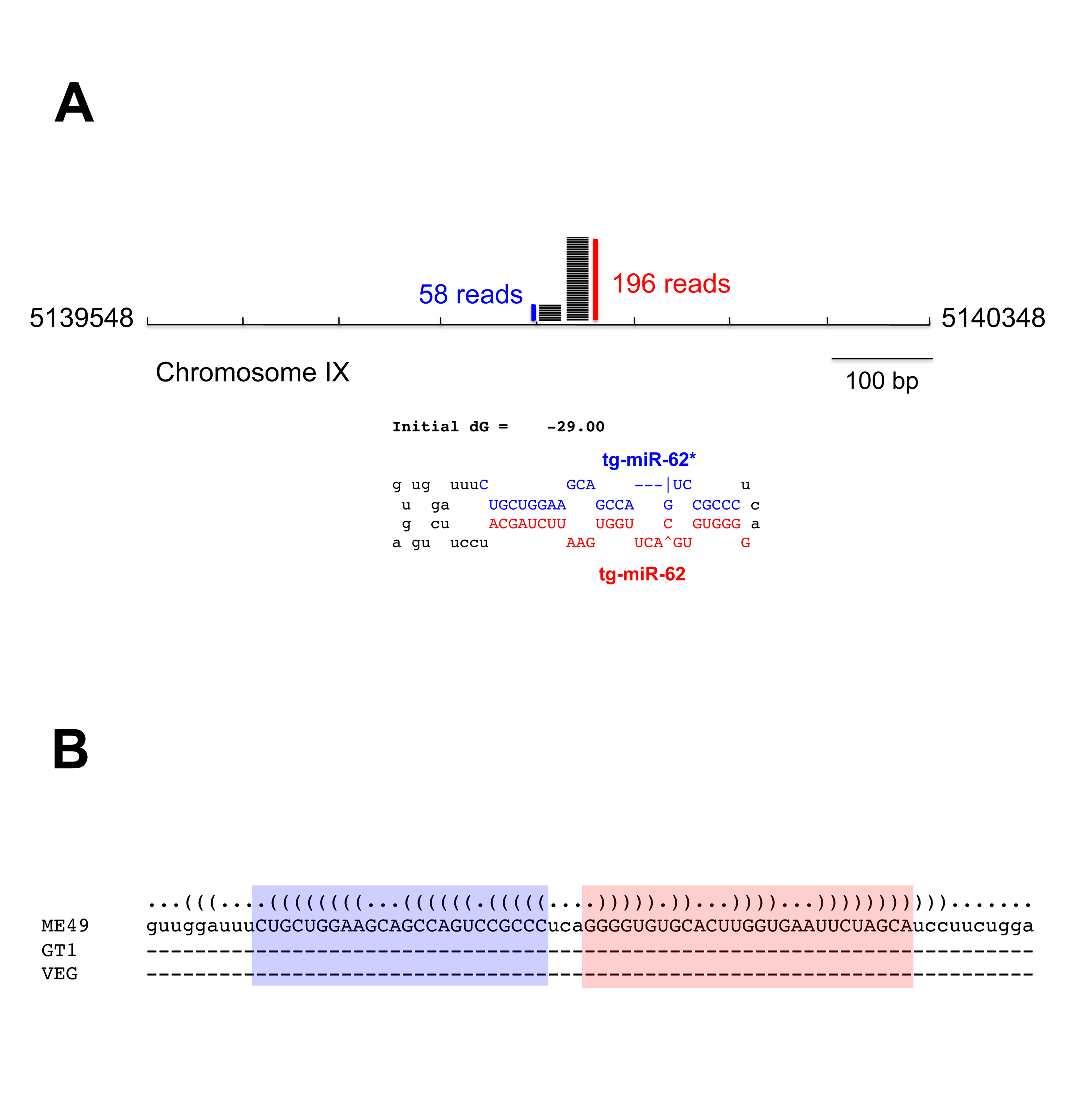

Supplement: Figure S10 — Characteristics of Tg-miR-62. (A) A miR-62 production hot spot in chromosome IX is shown along with the predicted structure and (B) the sequence conservation across parasite species. The mature region is shown in red and the passenger strand (microRNA*) in blue. Same legend as in Figure S2A. (0.46 MB TIF) [file ppat.1000920.s010.tif]

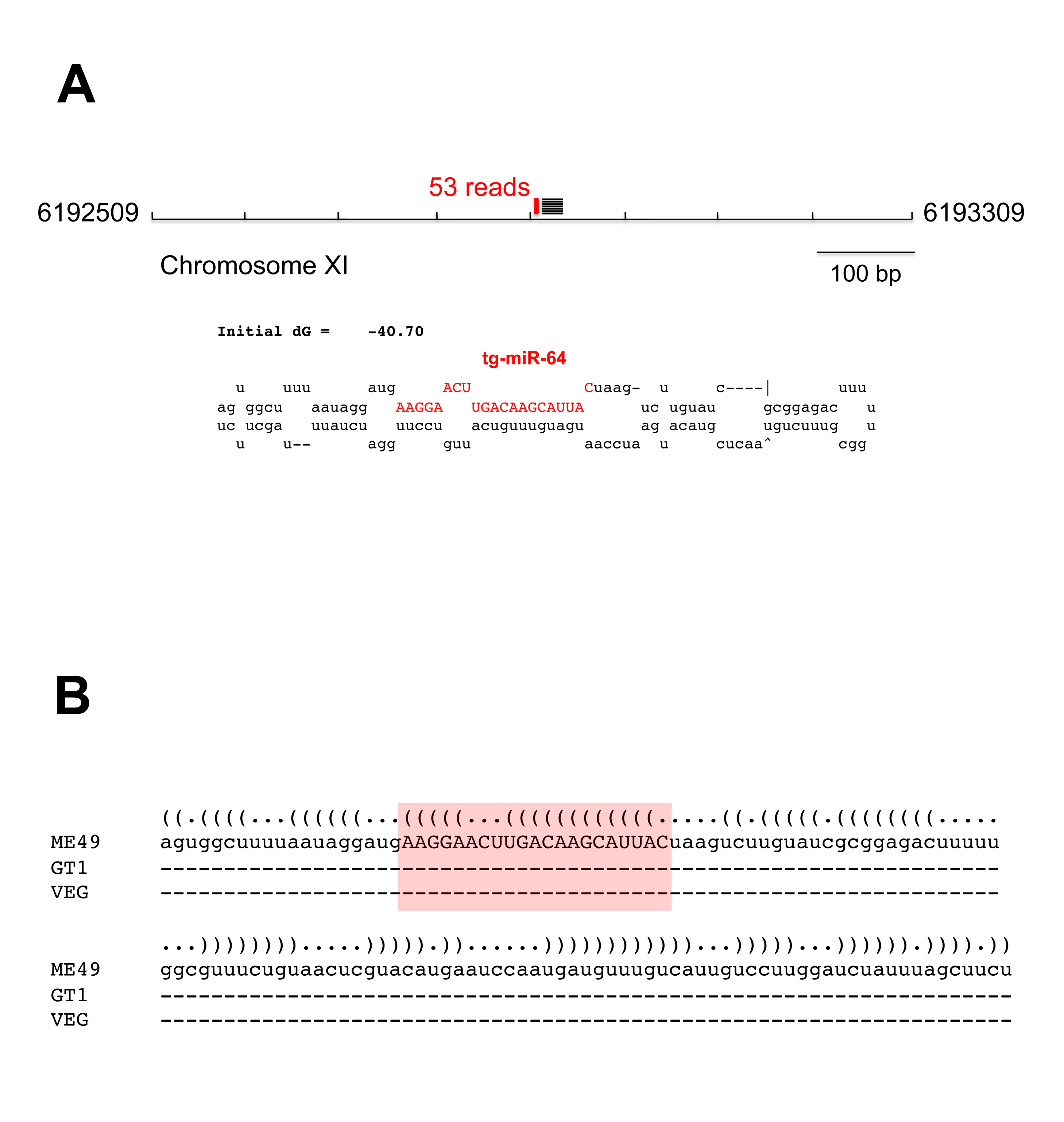

Supplement: Figure S11 — Characteristics of Tg-miR-64. (A) A miR-64 production hot spot in chromosome XI is shown along with the predicted structure and (B) the sequence conservation across parasite species. The mature region is shown in red. Same legend as in Figure S2A. (0.52 MB TIF) [file ppat.1000920.s011.tif]

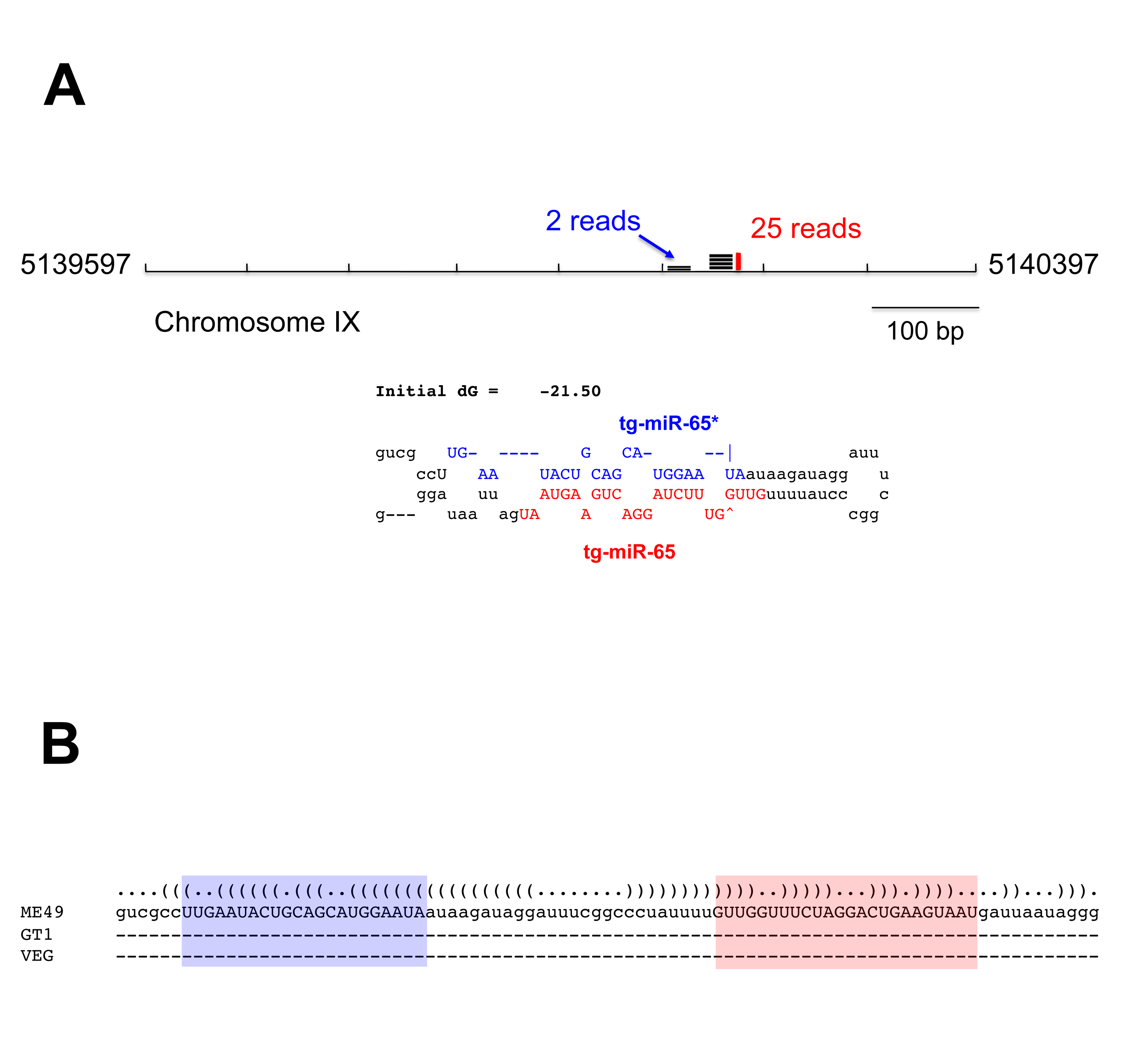

Supplement: Figure S12 — Characteristics of Tg-miR-65. (A) A miR-65 production hot spot in chromosome IX is shown along with the predicted structure and (B) the sequence conservation across parasite species. The mature region is shown in red and the passenger strand (microRNA*) in blue. Same legend as in Figure S2A. (0.45 MB TIF) [file ppat.1000920.s012.tif]

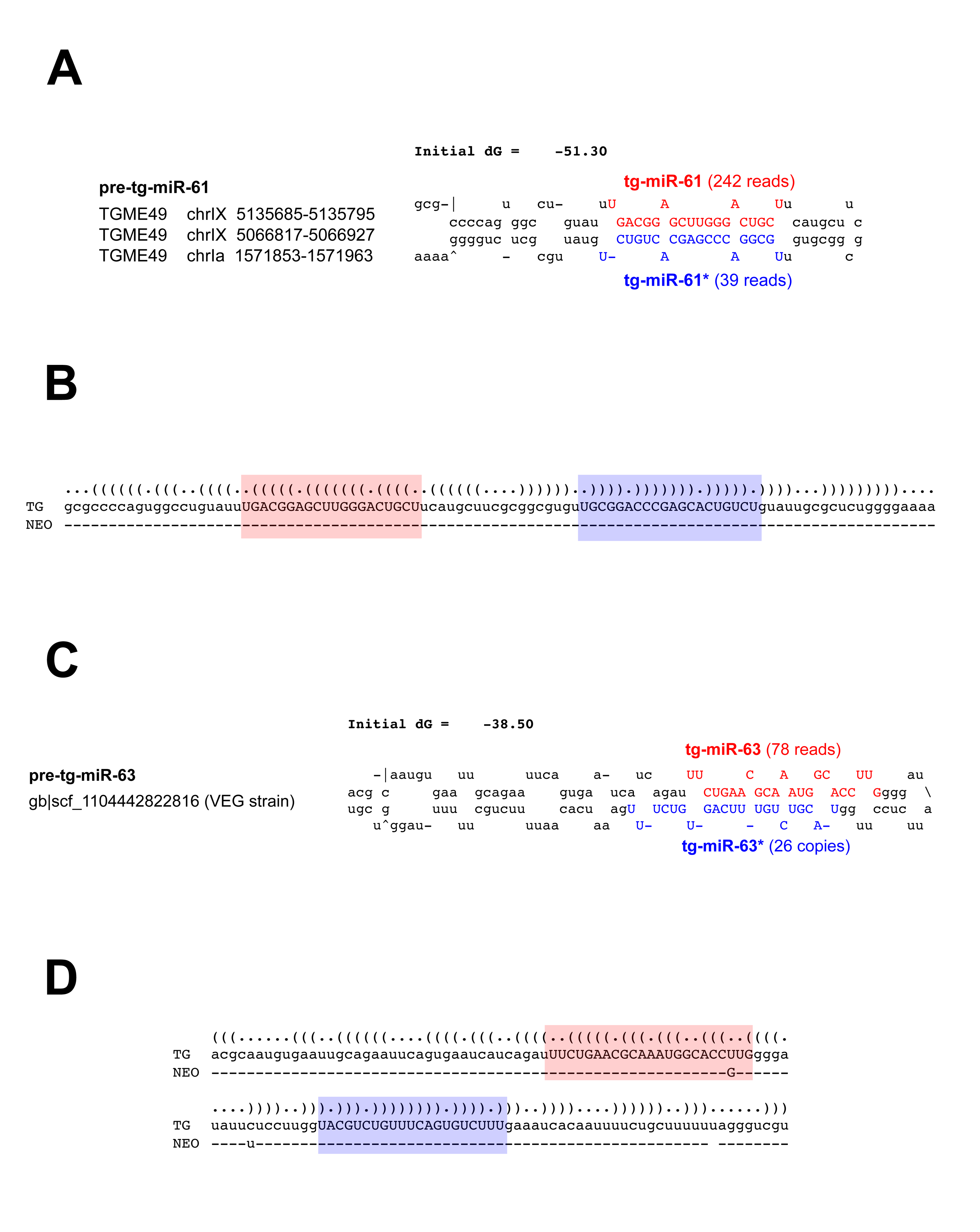

Supplement: Figure S13 — Characteristics of Tg-miR-61 and -63. (A) Predicted structure of miR-61 is shown along with (B) the sequence conservation across parasite species. The pre-miR-61 stem-loop structure is conserved across three loci on chromosomes IX and Ia. (C) Predicted structure of miR-63 is shown along with (D) the sequence conservation across parasite species. The mature region is shown in red and the passenger strand (microRNA*) in blue. Same legend as in Figure S2A. (0.79 MB TIF) [file ppat.1000920.s013.tif]

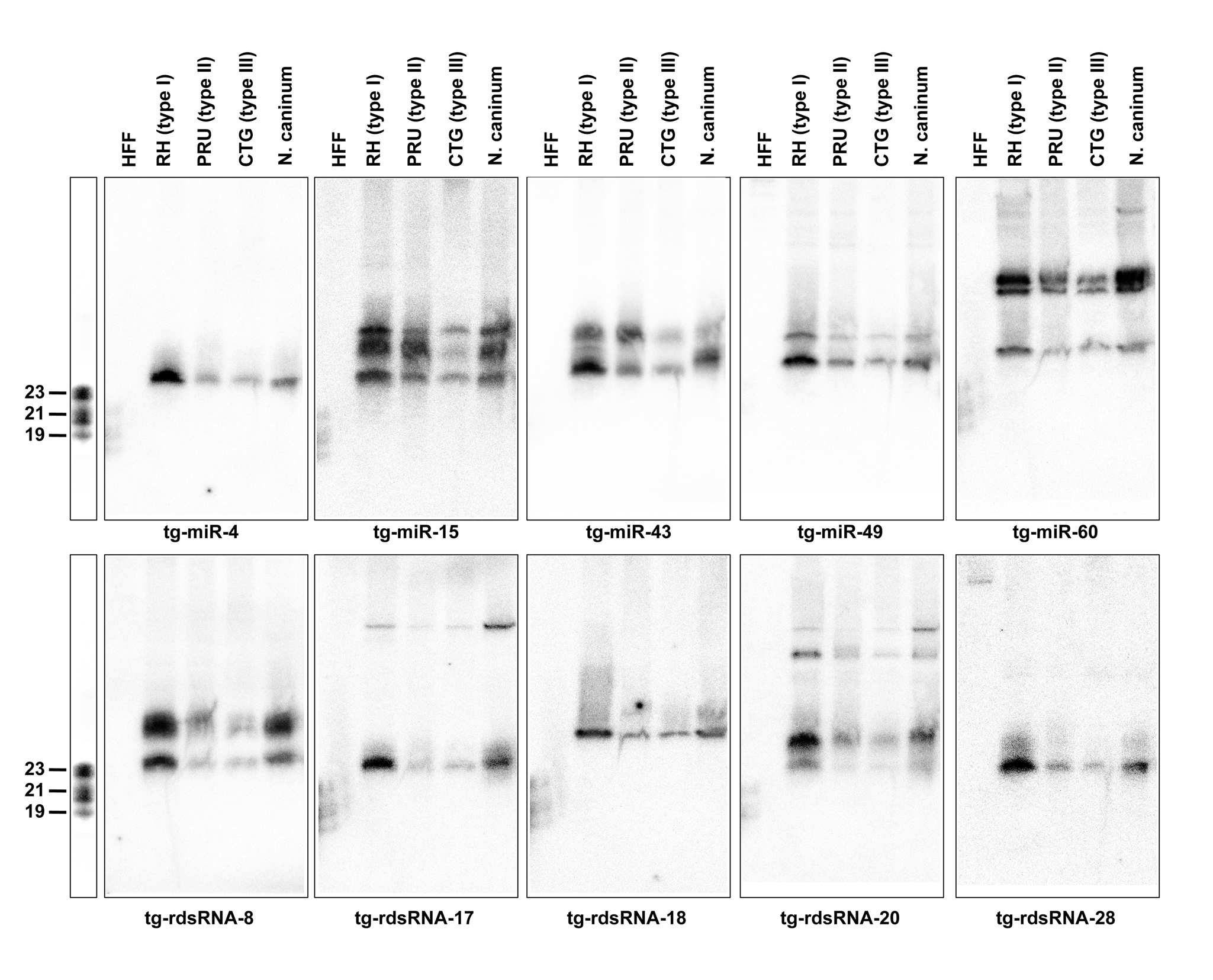

Supplement: Figure S14 — Northern analysis of Tg-miR and Tg-rdsRNAs. Full-size images of RNA blot phosphoimager scans used to generate panels A and B in Figures 4 and 5, respectively. Same legend as in Figures 2A and 3A. RNA markers (left lane) are 19, 21 and 23 nucleotides. (2.37 MB TIF) [file ppat.1000920.s014.tif]

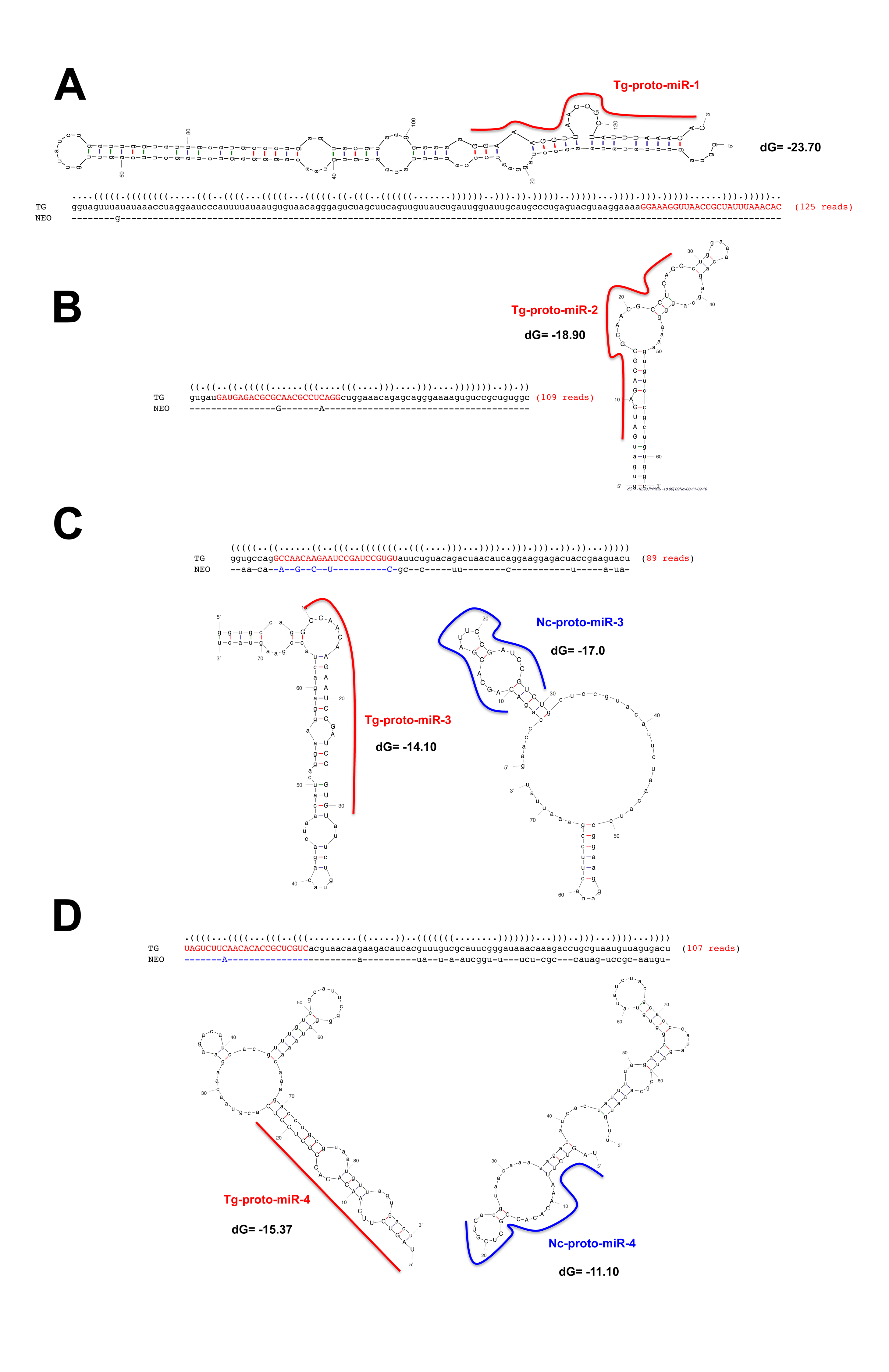

Supplement: Figure S15 — Identification of putative Toxoplasma proto-microRNAs. Secondary structure of T. gondii proto-miR-1 (A), -2 (B), -3 (C) and -4 (D) foldbacks compared to predicted secondary structure of the orthologous sequences from N. caninum. The red line indicates the cloned mature T. gondii miRNA sequence, while the blue line refers to the corresponding N. caninum sequence. Number of reads: Tg-proto-miR-1 (158 reads), Tg-proto-miR-2 (109 reads), Tg-proto-miR-3 (138 reads) and Tg-proto-miR-4 (107 reads). (0.93 MB TIF) [file ppat.1000920.s015.tif]

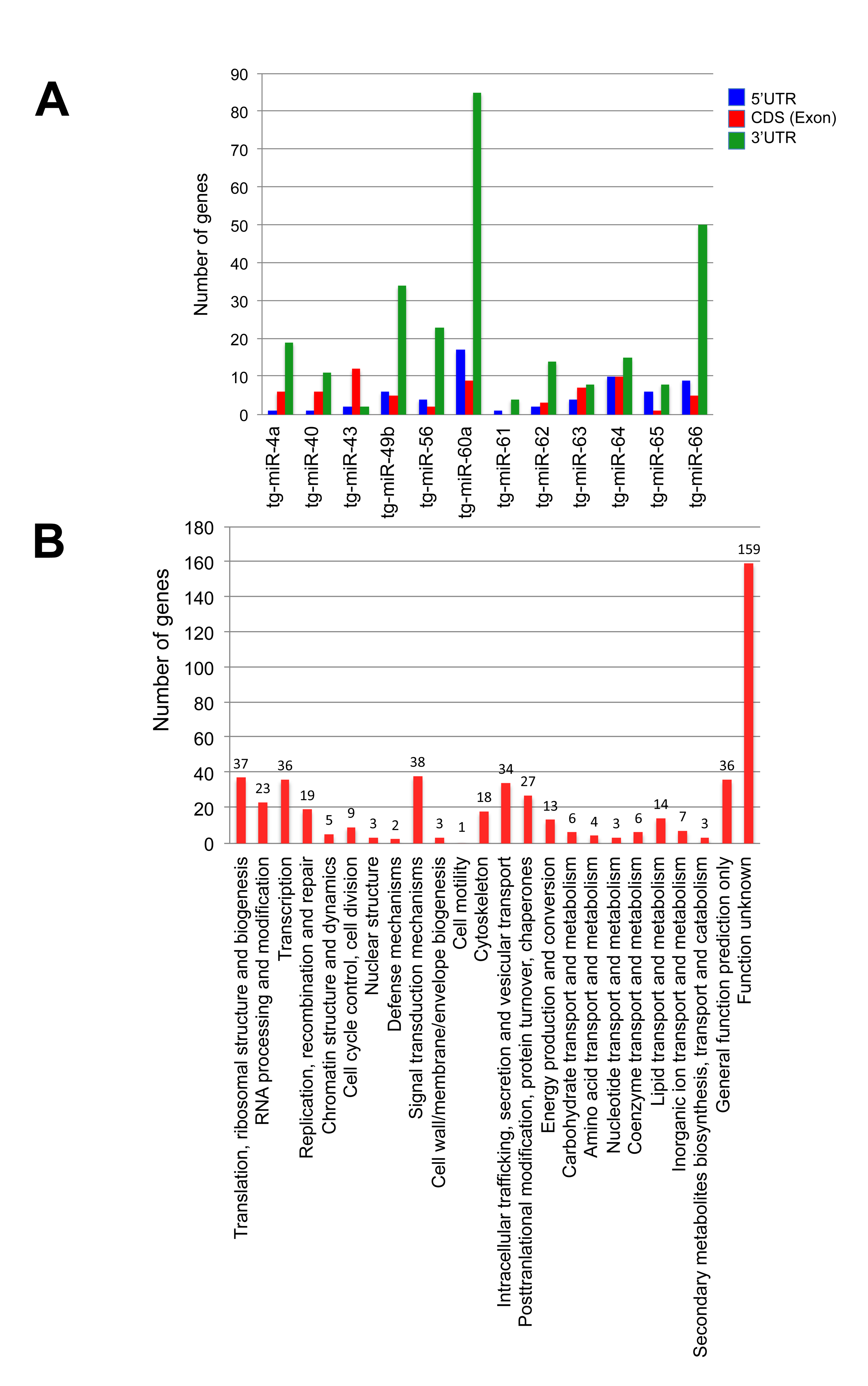

Supplement: Figure S16 — Prediction of Toxoplasma miRNA target genes. (A) The number of predicted target genes are shown for 12 Tg-microRNAs. (B) Genes targeted by the 14 Tg-miRNA families were functionally classified using the eukaryotic Clusters of Orthologous Groups (KOG) database. (1.07 MB TIF) [file ppat.1000920.s016.tif]
