# Supplementary material for: A Complex Small RNA Repertoire Is Generated by a Plant/Fungal-Like Machinery and Effected by a Metazoan-Like Argonaute in the Single-Cell Human Parasite Toxoplasma gondii
Source: PLoS Pathog. 2010 May 27;6(5):e1000920. doi: 10.1371/journal.ppat.1000920 (PMC2877743; doi:10.1371/journal.ppat.1000920)
Supplement: Table S1 — Toxoplasma tg-microRNAs. (0.03 MB PDF) [file ppat.1000920.s017.pdf]

Supplemental Table S1: *Toxoplasma* tg-microRNAs

tg-miR name	miR sequence	reads	lenght	miR* sequence	reads	lenght
tg-miR-4a	ATGTTTGCTTGGAAGCTGTAGTCATT	118571	26			
tg-miR-4b	ATGTTTGCTTGGAAGCCTTAGTCCTT	45411	26			
tg-miR-4c	ATGTTTGATTGGGAGCCTGAGTAGGT	467	26			
tg-miR-4d	ATGTTTGCTTGGAAGCTGTTGTACTT	355				
tg-miR-4e	ATGTTTGATTGGAAGCTGTTTTCCTT	351				
tg-miR-4f	ATGTTTGATTGGACGCTGTTTTCTTT	296				
tg-miR-4g	ATGTTTTATTGTACGCTGTCGTTATT	268				
tg-miR-4h	ATGTTTTCTTGGAATCCGTTGTCGTT	244				
tg-miR-15a	AAAGGGATGTTTAGCCGGGAAGCT	8697	24			
tg-miR-15b	ACAGGGATGGTTCTCCGGGAAGTT	2839	24			
tg-miR-15c	AAAGGGATGTTTAGCCGGGAAGTT	2275	24			
tg-miR-15d	AAAGGGAGGTTTAGCCTCGAATTT	2187	24			
tg-miR-15d	AAAGGGATGTTTAGCCGGGAAGTG	936	24			
tg-miR-24a	CCAGAGTGATGACCTTTGATGA	272	22	TTCAATTGAGGCATTTGTCTGAC	49	23
tg-miR-24b	CCAGAGTGATGACCTTTGATGA	272	22	TTCAATTGAGGCATTTGTCTGAC	49	23
tg-miR-40a	TTCGTTGACTCTGTTTACCACGGG	298	24			
tg-miR-40b	TCGTTGACTGTGTTTACCACGGG	190	23			
tg-miR-43	AGTTCTATTGTGTTAGCATGGTTTC	8610	25			
tg-miR-49a	CGGGGAGAAGGGCAGTGCGTTT	170607	22			
tg-miR-49b	CGGCGAGAAGGGAAGTGT	18806	18			
tg-miR-56	GGAUGACCCAGGAAGUCCCAGCUCAC	5	26			
tg-miR-60a	ACACAGTCGGTACGAAATCCATACT	280723	25	AATTGATTATGCTGACTTTAGTC	3	23
tg-miR-60b	ACACAGTCGGTACGAAGTCGAAACA	25243	25			
tg-miR-60c	ACACATGCGGTATGAAGGCTAAACA	10220	25			
tg-miR-60d	ACACAGGCGGTACGAACGGTTAACA	9921	25			
tg-miR-60e	ACACAGACGGTGCGGGGTCGAAACC	3145	25			
tg-miR-60f	ACACAGACGGTACGAAATCGAAAC	2622	24			
tg-miR-60g	ACACAGACGCGACGAAGTCGAAACG	1633	25			
tg-miR-60h	ACACAGACGGGACTAAGCCGTAACA	1507	25			
tg-miR-61	TGACGGAGCTTGGGACTGCT	242	20	TGCGGACCCGAGCACTGTCT	39	20
tg-miR-62	GGGGTGTGCACTTGGTGAATTCTAGCA	196	27	CTGCTGGAAGCAGCCAGTCCGCCC	58	24
tg-miR-63	TTCTGAACGCAAATGGCACCTTG	78	23	TACGTCTGTTTCAGTGTCTTT	26	21
tg-miR-64	AAGGAACTTGACAAGCATTAC	53	21			
tg-miR-65	GTTGGTTTCTAGGACTGAAGTAAT	25	24	TTGAATACTGCAGCATGGAATA	2	22
tg-miR-66	GGGGAAGGTAGGCGAAGCGGTC	18	22	CGACCGCTTCGTTTACCTTTCC	3	22
