# Supplementary material for: A Complex Small RNA Repertoire Is Generated by a Plant/Fungal-Like Machinery and Effected by a Metazoan-Like Argonaute in the Single-Cell Human Parasite Toxoplasma gondii
Source: PLoS Pathog. 2010 May 27;6(5):e1000920. doi: 10.1371/journal.ppat.1000920 (PMC2877743; doi:10.1371/journal.ppat.1000920)
Supplement: Table S2 — Predicted binding sites of selected T. gondii tg-miRNA. (0.04 MB PDF) [file ppat.1000920.s018.pdf]

Supplemental Table S2: Predicted binding sites of selected *T. gondii* tg-miRNA

Tg-miRNA	TARGET gene (TOXODB v4.3)	Gene location	mfe (Kcal/mol)		target genes mRNA expression RH / PRU strains (deltaCt)
miR-4a	20.m07347	3'UTR	-49,3	target 5' U G 3'<br>AAUGACUACAGCUUCCAAGCAAACAU<br>UUACUGAUGUCGAAGGUUCGUUUGUA<br>miRNA 3' 5'	9,057 / 8,913
miR-4a	50.m03142	3'UTR	-41,5	target 5' C CC U 3'<br>AGUGAC CAGCUUCCAAGCAAACAU<br>UUACUG GUCGAAGGUUCGUUUGUA<br>miRNA 3' AU 5'	
miR-4a	59.m03385	3'UTR	-38,9	target 5' U A U G 3'<br>AAUGA UACAGCUUCC AGCGAACAU<br>UUACU AUGUCGAAGG UCGUUUGUA<br>miRNA 3' G U 5'	6,955 / 7,022
miR-4a	42.m03635	CDS	-39,7	target 5' C C U G 3'<br>AAUGGCU CAGCUUCCAAGCG AGCGU<br>UUACUGA GUCGAAGGUUCGU UUGUA<br>miRNA 3' U 5'	5,988 / 6,017
miR-4a	80.m00082	CDS	-40,3	target 5' G G C 3'<br>GACUG AGCUUCCAAGCGGGCGU<br>CUGAU UCGAAGGUUCGUUUGUA<br>miRNA 3' UUA G 5'	
miR-40	33.m01361	3'UTR	-49	target 5' C U 3'<br>CCCGUGGUAAACACAGUCAACGAA<br>GGGCACCAUUUGUGUCAGUUGCUU<br>miRNA 3' 5'	5,180 / 5,462
miR-40	583.m09143	3'UTR	-44	target 5' U U 3'<br>CCCGUGGUAAACACAGUCAAC<br>GGGCACCAUUUGUGUCAGUUG<br>miRNA 3' CUU 5'	5,404 / 5,844
miR-40	583.m05712	CDS	-42,2	target 5' C G U 3'<br>CCCGUGG AAACAUAGUCAACGAG<br>GGGCACC UUUGUGUCAGUUGCUU<br>miRNA 3' A 5'	
miR-43	46.m01606	5'UTR	-42,5	target 5' U G 3'<br>AAACCGUGCUAGCACAGUAGAACU<br>UUUGGUACGAUUGUGUUAUCUUGA<br>miRNA 3' C 5'	4,136 / 4,334
miR-43	38.m02365	3'UTR	-44,6	target 5' A U 3'<br>GAAACCAUGCUAACACAAUAGAACU<br>CUUUGGUACGAUUGUGUUAUCUUGA<br>miRNA 3' 5'	
miR-43	42.m00077	3'UTR	-32,8	target 5' U C C C 3'<br>GAG CCAUGCUA CACAGUGGAAU<br>CUU GGUACGAU GUGUUAUCUUG<br>miRNA 3' U U A 5'	
miR-49b	49.m03305	CDS	-35,1	target 5' C C 3'<br>ACACUUCUUUUCUCGCCG<br>UGUGAAGGGAAGAGCGGC<br>miRNA 3' 5'	
miR-49b	57.m03990	3'UTR	-39	target 5' G U 3'<br>ACGCUUCCCUUCUCGCC<br>UGUGAAGGGAAGAGCGG<br>miRNA 3' C 5'	6,778 / 6,520
miR-49b	80.m02222	3'UTR	-35,9	target 5' C U 3'<br>ACACUUCCUUUCUCGCUG<br>UGUGAAGGGAAGAGCGGC<br>miRNA 3' 5'	
miR-49b	44.m02663	CDS	-34,2	target 5' G G U 3'<br>GCAUUU CCUUCUCGCCG<br>UGUGAA GGAAGAGCGGC<br>miRNA 3' G 5'	
miR-60b	55.m04980	3'UTR	-42	target 5' A C C 3'<br>GUUUCGACUUCG GCUGACUGUG<br>CAAAGCUGAAGC UGGCUGACAC<br>miRNA 3' A A A 5'	10,036 / 9,983
miR-60b	49.m03142	3'UTR	-32,8	target 5' C A A U 3'<br>UUUCGGCU CGUAUCGACU UGU<br>AAAGCUGA GCAUGGCUGA ACA<br>miRNA 3' AC A C 5'	
miR-60b	42.m03570	5'UTR	-25,8	target 5' U AC U C 3'<br>GUUUCGACUUCGCAC ACU UGU<br>CAAAGCUGAAGCGUG UGA ACA<br>miRNA 3' A GC C 5'	
miR-60b	20.m03918	CDS	-50,3	target 5' G U 3'<br>UGUUUCGACUUCGUACCGACUGUGU<br>ACAAAGCUGAAGCAUGGCUGACACA<br>miRNA 3' 5'	

miR-61 33.m01324 CDS -33,2

```
target 5' U          A  G    U 3'
           GCAGUCCCAA CU CGUC
           CGUCAGGGUU GA GCAG
miRNA  3' U          C  G    U 5'
```

miR-61 52.m01583 CDS -36,4

```
target 5' C       A             G 3'
           AGCAG C CCAAGCUCUGUCA
           UCGUC G GGUUCGAGGCAGU
miRNA  3'       A                 5'
```

miR-62 46.m01648 3'UTR -30,9

```
target 5' A            G   G         C 3'
           GC   GAUUCAC AAG GCACACUUU
           CG   UUAAGUG UUC CGUGUGGGG
miRNA  3' A  AUC        G   A           5'
```

miR-62 44.m02773 CDS -39

```
target 5' G  C           U         G  3'
           GC AGAAUUCAUCA GUGCACGCU
           CG UCUUAAGUGGU CACGUGUGG
miRNA  3' A  A           U         GG 5'
```

miR-63 72.m00399 CDS -30,4

```
target 5' C   CA          U       C 3'
           CAA  UGCCGUUUGC UUCAGAA
           GUU  ACGGUAAACG AAGUCUU
miRNA  3'     CC          C         5'
```

miR-63 44.m02738 CDS -32,3

```
target 5'   U  CA               C   U 3'
             GG  UGCCGUUUGCGUUCA GGA
             CC  ACGGUAAACGCAAGU CUU
miRNA  3' GUU                         5'
```

miR-64 583.m05724 3'UTR -36,9

```
target 5' A                    G 3'
           UAAUGCUUGUCAAGUUCCUU
           AUUACGAACAGUUCAAGGAA
miRNA  3' C                      5'
```

miR-64 80.m02148 CDS -32,9

```
target 5'  A                   G 3'
            AAUGCUUGUCAAGUUUCUU
            UUACGAACAGUUCAAGGAA
miRNA  3' CA                     5'
```

miR-64 541.m00125 5'UTR -31,7

```
target 5' A          C          G 3'
           GUAAUGCUUG UAAGUUCCUU
           CAUUACGAAC GUUCAAGGAA
miRNA  3'            A            5'
```

miR-64 59.m00060 CDS -31,7

```
target 5' C    G                A 3'
           GUGA GUUUGUCGAGUUCCUU
           CAUU CGAACAGUUCAAGGAA
miRNA  3'      A                  5'
```

miR-66 38.m01033 5'UTR -53,9

```
target 5' C                      U 3'
           GACCGCUUCGCCUACCUUCCCC
           CUGGCGAAGCGGAUGGAAGGGG
miRNA  3'                          5'
```

miR-66 50.m03243 CDS -37,9

```
target 5'   A                   U 3'
             UGCUUCGCCUGCCUUCUUU
             GCGAAGCGGAUGGAAGGGG
miRNA  3' CUG                     5'
```

miR-66 52.m01615 CDS -42,5

```
target 5'  G                   G 3'
            UCGCUUCGUCUGCCUUCCC
            GGCGAAGCGGAUGGAAGGG
miRNA  3' CU                   G 5'
```

miR-66 35.m01593 CDS -37,3

```
target 5'  U              A   G  3'
            CUGCUUCGCCUACC UCC
            GGCGAAGCGGAUGG AGG
miRNA  3' CU              A   GG 5'
```

miR-66 55.m04934 3'UTR -36,7

```
target 5' C  G       G C        A 3'
           AC GCUUCGC U UCUUCCCC
           UG CGAAGCG A GGAAGGGG
miRNA  3' C  G       G U          5'
```
