# Supplementary material for: A Complex Small RNA Repertoire Is Generated by a Plant/Fungal-Like Machinery and Effected by a Metazoan-Like Argonaute in the Single-Cell Human Parasite Toxoplasma gondii
Source: PLoS Pathog. 2010 May 27;6(5):e1000920. doi: 10.1371/journal.ppat.1000920 (PMC2877743; doi:10.1371/journal.ppat.1000920)
Supplement: Table S3 — Toxoplasma REP-derived small RNAs (rdsRNAs). (0.03 MB PDF) [file ppat.1000920.s019.pdf]

Supplemental Table S3: *Toxoplasma* REP-derived small RNAs (rdsRNAs)

Name	Sequence	reads	lenght	REP
tg-rdsRNA-8	CTTCTTGGCATAACGTTGTGTAG	1155	23	REP3 (antisense)
tg-rdsRNA-17	TGTAACGGTACAAGCTGTAAA	464	21	REP2 (sense)
tg-rdsRNA-18	GATCATCGAATATAACGGTATGCTCCTG	11344	28	REP2 (sense)
tg-rdsRNA-19	ACAGGATCAAATCTTCCTTGAGCGAC	1326	26	REP3 (antisense)
tg-rdsRNA-20	TGATTGGTATTGCATGCCTGGTGAC	1066	25	REP1 (antisense)
tg-rdsRNA-28	CTAAAGATAGCGTGAAAGCTC	223	21	REP3 (antisense)
candidate 1	GATCTTGAAGGTCTTTGTTTACCGGATCC	5	29	SDR
candidate 2	ACCTTCAAGATCTAAACCA	3	19	SDR
candidate 3	AGTCCAACTCGAATTATATACTCCCC	3	26	SDR
