# Supplementary material for: A Complex Small RNA Repertoire Is Generated by a Plant/Fungal-Like Machinery and Effected by a Metazoan-Like Argonaute in the Single-Cell Human Parasite Toxoplasma gondii
Source: PLoS Pathog. 2010 May 27;6(5):e1000920. doi: 10.1371/journal.ppat.1000920 (PMC2877743; doi:10.1371/journal.ppat.1000920)
Supplement: Table S4 — Summary of Tg-AGO-associated proteins identified by mass spectrometry. (0.12 MB PDF) [file ppat.1000920.s020.pdf]

Supplemental Table S4 : Summary of *Tg*-AGO-associated proteins identified by mass spectrometry

T. gondii proteins homologous of known argonaute interactors in human or *Drosophila melanogaster* (D.m.) are highlighted in blue (those implicated in translational control and RNA metabolism) or in red (those implicated in heterochromatin formation or transcriptional control). MS : mass spectrometry.

MS Band	ToxoDB v4.3 ID	# Peptides	Human Homolog (GeneID)	PFAM and SMART Domains	REF
3	83.m01262	24	DDX60L (91351)	Helicase / DEAD box	
3	583.m05616	8	AQR (9716)	Intron-binding protein aquarius	
3	42.m03275	4	ASCC3L1 (23020)	Helicase / DEAD box	
4	641.m01480	3	DHX57 (90957)	Helicase / DEAH box	
1b	44.m02690	4	DHX16 (8449)	Helicase / DEAH box	
10a	44.m02802	9	DHX15 (1665)	Helicase / DEAH box	
**11**	**42.m00117**	**18**	**DDX3X (1654) - Bel (D.m.)**	**Helicase / DEAD box**	**40, 68**
**14**	**46.m00027**	**9**	**DDX17 (10521)**	**Helicase / DEAD box, DDX5-like**	**39, 40**
**16**	**35.m00026**	**20**	**DDX39 / BAT1 (7919)**	**Helicase / DEAD box**	**39**
**15**	**583.m00676**	**3**	**DDX6 /RCK/p54 (1656)**	**Helicase / DEAD box**	**67**
**18**	**55.m04649**	**5**	**DDX48 (9775)**	**Helicase / DEAD box**	**39**
**9a**	**35.m00901**	**53**	**FUBP2/ KSRP (8570)**	**KH**	**40**
**12b**	**46.m00023**	**15**		**KH, FMR/FXR-like**	**39, 67**

**5**	**25.m02940**	**9**		**KH, FMR/FXR-like**	**39, 67**
**10a**	**49.m00009**	**7**	**SND1/p100 (27044)**	**tudor / staphylococcal nuclease**	**65**
**10a**	**42.m03384**	**20**	**PABPC1 (26986)**	**PABC, RRM, PABPC4-like**	**39, 40**
**9a**	**80.m02340**	**17**	**Nucleolin (4691)**	**RRM**	**39**
**15**	**57.m01690**	**7**	**HNRNPA3 (220988)**	**RRM**	**40**
**15**	**46.m01699**	**14**	**HNRPH1 (3187)**	**RRM, HNRPF-like**	**39, 40**
**21b**	**55.m00241**	**34**	**HNRNPM (4670)**	**RRM**	**40**
**15**	**80.m00057**	**5**	**HNRNPL (3191)**	**RRM**	**39, 40**
**13a**	**50.m00035**	**4**	**NOP56 (10528)**	**Nucleolar protein**	**39**
13b	20.m00365	14	NOP58 (51602)	Nucleolar protein	
11	59.m06060	25	LARP2 (55132)	HTH La-type RNA-binding	
21b	TgTwinScan_0277	5		RRM	
10b	80.m02342	4	U2AF2 (11338)	RRM	
7	83.m01280	3		RRM	
20	645.m00319	12		RRM, WW	
15	645.m00330	12		nuclear RNA binding protein	
**20**	**55.m00015**	**25**	**14-3-3 family**	**14-3-3 domain**	**69, 71**
10b	49.m03354	6	PRPF4B (8899)	Serine/threonine-protein kinase	
20	35.m00002	8	RACK1 (10399)	receptor for activated C kinase	

1b	44.m02663	17	GCN1L1 (10985)	Translational activator	
21a	20.m03918	23	NACA (4666)		
**9c**	**20.m03912**	**39**	**EEF2 (1938)**	**translation elongation factor**	**40**
**15**	**76.m00016**	**42**	**EEF1A1 (1915)**	**translation elongation factor**	**40**
18	145.m00003	5	EEF1G (1937)	translation elongation factor	
20	42.m00069	13	EEF1B2 (1933)	translation elongation factor	
21b	38.m01892	4	EEF1D (1936)	translation elongation factor	
9c	59.m03731	6	EIF2B5 (8893)	translation initiation factor	
10a	41.m00006	4	EIF3B (8662)	translation initiation factor	
11	59.m00055	50	EIF3EIP (51386)	translation initiation factor	
12b	611.m00038	18	EIF3D (8664)	translation initiation factor	
13a	59.m06091	6	EIF3E (3646)	translation initiation factor	
16	50.m03396	16	EIF3H (8667)	translation initiation factor	
16	46.m00024	9	EIF2S3 (1968)	translation initiation factor	
18	583.m00670	6	EIF2S1 (1965)	translation initiation factor	
20	80.m02245	4	EIF3I (8668)	translation initiation factor	
20	46.m00016	8	EIF2S2 (8894)	translation initiation factor	
**21b**	**42.m03276**	**6**	**EIF4E2 (9470)**	**translation initiation factor**	**67**
21a	55.m04970	12	EIF3K (27335)	translation initiation factor	

16	50.m05680	41	EIF4A1 (1973)	translation initiation factor	
**20**	**38.m00002**	**7**	**RPLP0 (6175)**	**60S acidic ribosomal protein P0**	**40**
**18**	**42.m00084**	**8**	**RPL3 (6122)**	**60s ribosomal protein L3**	**40**
**18**	**583.m00619**	**18**	**RPL4 (6124)**	**60s ribosomal protein L4**	**40**
**19**	**641.m00186**	**10**	**RPL5 (6125)**	**60S ribosomal protein L5**	**40**
**21b**	**583.m05552**	**9**	**RPL6 (6128)**	**60s ribosomal protein L6**	**40**
**21b**	**583.m00012**	**7**	**RPL7 (6129)**	**60S ribosomal protein L7**	**40**
**20**	**55.m00280**	**5**	**RPL7A (6130)**	**60S ribosomal protein L7a**	**40**
**21a**	**20.m00388**	**11**	**RPL8 (6132)**	**60S ribosomal protein L8**	**40**
**21b**	**33.m01368**	**16**	**RPL10a (4736)**	**60S ribosomal protein L10a**	**40**
**21b**	**55.m00189**	**8**	**RPL13 (6137)**	**60S ribosomal protein L13**	**40**
**19**	**57.m00025**	**6**	**RPSA (3921)**	**40S ribosomal protein SA**	**40**
**21a**	**541.m00133**	**16**	**RPS2 (6187)**	**40S ribosomal protein S2**	**40**
**21a**	**44.m04669**	**21**	**RPS3 (6188)**	**40S ribosomal protein S3**	**40**
**20**	**44.m02744**	**17**	**RPS3A (6189)**	**40S ribosomal protein S3A**	**40**
**21a**	**25.m00221**	**12**	**RPS4X (6191)**	**40S ribosomal protein S4**	**40**
**21a**	**27.m00119**	**11**	**RPS6 (6194)**	**40s ribosomal protein S6**	**40**
**21b**	**49.m00013**	**9**	**RPS7 (6201)**	**40S ribosomal protein S7**	**40**
**21c**	**50.m00067**	**18**	**RPS8 (6202)**	**40S ribosomal protein S8**	**40**

4	TGME49_105340	14		TgCRC230	75
9c	583.m00590	5	TBL1 (6907)	LISH, ANK	75
**16**	**42.m00014**	**4**	**HDAC1 (3065)**	**TgHDAC3**	**74, 75**
3	583.m05395	3		AP2 DNA binding domain	
5	641.m01573	4	SMARCA2 (6595)	SWI/SNF-related	
18	38.m00018	4	PRMT1	TgPRMT1	75
9c	55.m05071	4		ANK, BROMO	
3	583.m05259	4	SPT6 (6830)	Transcription elongation factor	
12a	55.m04934	5	SSRP1 / FACT80 (6749)	transcriptional elongation factor	
**8**	**55.m04747**	**3**	**RPB2 (5431)**	**DNA-directed RNA polymerase II**	**72**
19	50.m00004	6	PCNA (5111)	proliferating cell nuclear antigen	
19	50.m03067	4	BUB3 (9184)	WD repeat	
14	50.m03261	6		single-strand DNA-binding protein	
20	41.m03144	4		Alba domain	
17	49.m00035	7		putative S1/P1 endonuclease	
16	69.m00140	16	PA2G4 (5036)	Proliferation-associated protein 2G4	
9c	49.m00023	3	PES1 (23481)	BRCT, Pescadillo-like	
